# The role of parametric feature maps to correct different volume of interest sizes: an *in vivo* liver MRI study

**DOI:** 10.1186/s41747-023-00362-9

**Published:** 2023-09-06

**Authors:** Laura Jacqueline Jensen, Damon Kim, Thomas Elgeti, Ingo Günter Steffen, Lars-Arne Schaafs, Bernd Hamm, Sebastian Niko Nagel

**Affiliations:** Charité–Universitätsmedizin Berlin, Department of Radiology, Corporate Member of Freie Universität Berlin, Humboldt-Universität zu Berlin, Berlin Institute of Health, Hindenburgdamm 30, 12203 Berlin, Germany

**Keywords:** Magnetic resonance imaging, Liver, Radiomics, Reproducibility of results, Software

## Abstract

**Background:**

Different volume of interest (VOI) sizes influence radiomic features. This study examined if translating images into feature maps before feature sampling could compensate for these effects in liver magnetic resonance imaging (MRI).

**Methods:**

T1- and T2-weighted sequences from three different scanners (two 3-T scanners, one 1.5-T scanner) of 66 patients with normal abdominal MRI were included retrospectively. Three differently sized VOIs (10, 20, and 30 mm in diameter) were drawn in the liver parenchyma (right lobe), excluding adjacent structures. Ninety-three features were extracted conventionally using PyRadiomics. All images were also converted to 93 parametric feature maps using a pretested software. Agreement between the three VOI sizes was assessed with overall concordance correlation coefficients (OCCCs), while OCCCs > 0.85 were rated reproducible. OCCCs were calculated twice: for the VOI sizes of 10, 20, and 30 mm and for those of 20 and 30 mm.

**Results:**

When extracted from original images, only 4 out of the 93 features were reproducible across all VOI sizes in T1- and T2-weighted images. When the smallest VOI was excluded, 5 features (T1-weighted) and 7 features (T2-weighted) were reproducible. Extraction from parametric maps increased the number of reproducible features to 9 (T1- and T2-weighted) across all VOIs. Excluding the 10-mm VOI, reproducibility improved to 16 (T1-weighted) and 55 features (T2-weighted). The stability of all other features also increased in feature maps.

**Conclusions:**

Translating images into parametric maps before feature extraction improves reproducibility across different VOI sizes in normal liver MRI.

**Relevance statement:**

The size of the segmented VOI influences the feature quantity of radiomics, while software-based conversion of images into parametric feature maps before feature sampling improves reproducibility across different VOI sizes in MRI of normal liver tissue.

**Key points:**

• Parametric feature maps can compensate for different VOI sizes.

• The effect seems dependent on the VOI sizes and the MRI sequence.

• Feature maps can visualize features throughout the entire image stack.

**Graphical Abstract:**

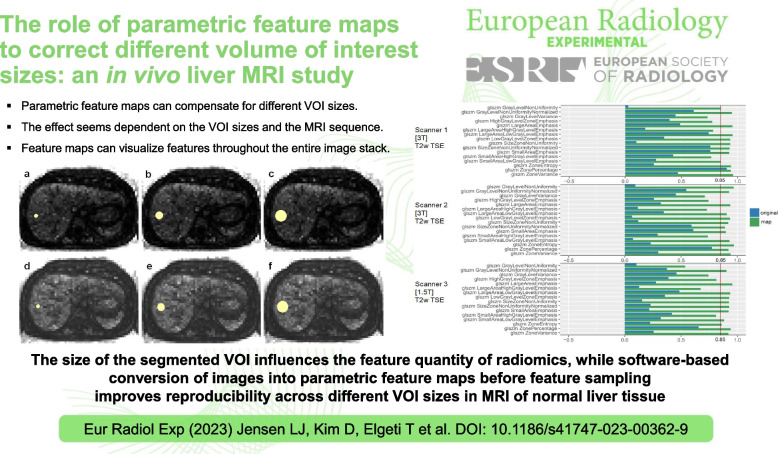

**Supplementary Information:**

The online version contains supplementary material available at 10.1186/s41747-023-00362-9.

## Background

To gain high-dimensional data invisible to the human eye from radiological images with the “radiomics” approach and use these as quantitative imaging biomarkers appears promising [1, 2]. In this process, multiple quantitative features, based on texture, intensity, shape, and size, are extracted from digital images aiming to develop decision-supporting tools in medicine [2]. For example, a recent study found a correlation between textural features of mice livers with intrahepatic tumor growth after injecting colon cancer cells before the metastases became visible to the human eye [3]. Another group could predict malignancy in fat-suppressed T1-weighted magnetic resonance imaging (MRI) sequences of soft tissue tumors [4]. A growing body of other studies showed correlations between feature behavior and different diagnostic endpoints such as tumor biology, tumor response, or therapy response [3, 5–7].

A translational gap exists between evolving scientific results and the still lacking application of radiomics in clinical practice [8]. Poor reproducibility is the primary limitation of introducing radiomics into the clinical routine [8–10]. Both the intrinsic properties of the images, like different acquisition parameters, reconstruction algorithms, image noise, and resolution, and the postprocessing steps, like segmentation and applied software, affect feature reproducibility [11–14]. In particular, also the size of the segmented volume of interest (VOI) influences feature quantity [15–19]. Phantom and *in vivo* studies corroborated that differences in VOI size could cause significantly different results for many features, conceivably falsifying links between radiomics and biological endpoints [15, 16].

A recent phantom study proposed preprocessing radiological images into parametric feature maps to reduce the confounding effects of different VOI sizes [20]. Parametric feature maps can be created with a pretested software tool that computes the whole image stack into a feature map stack. Separate feature maps are calculated for every feature analyzed. More precisely, the software divides the image into voxels of a defined voxel size so that all features are calculated for small, equally sized VOIs (*i.e.*, the voxels). The resulting feature quantities are stored in the maps, where the gray levels reflect the quantity of the feature, *e.g.,* regions in the maps with high quantities for the respective feature appear bright. Feature values can then be directly retrieved from the map in the same manner as Hounsfield units in a standard image viewer [21].

The aim of this study was to evaluate if parametric maps can correct for different VOI sizes in MRI of nonpathological livers.

## Methods

### Study group

The patient group was already included in a previous study with the approval of the institutional review board [EA1/104/19] [16]. The study group comprised 66 patients examined in clinical routine between April 2012 and August 2020 to exclude a chronic inflammatory bowel disease. Examinations from three different MRI scanners were included. Only patients without disease were considered (*i.e.,* the patient's record had to be devoid of disorders). Also, patients with liver lesions or parenchymal abnormalities (*e.g.,* signal alteration between in-phase and opposed phase) and patients with metal implants (*e.g.,* dorsal instrumentation or hip replacement) were excluded to avoid disturbing factors. Details of the patient population are summarized in Table 1.

**Table 1:** Details on the study population

Scanner number	1	2	3
Patients	25	19	22
Sex (female/male)	15/10	13/6	14/8
Age (years): mean (range)	34.3 (17–62)	28.1 (15–49)	30.9 (15–49)

### MRI scanners and examination

Examinations were retrospectively screened from three different scanners: two 3-T scanners of the same model (Magnetom Skyra, Siemens Healthineers, Erlangen, Germany) and one 1.5-T scanner (Magnetom Aera, Siemens Healthineers, Erlangen, Germany). All scanners were calibrated regularly. The patients fasted for 4 h before the examination and were examined for 40 min with MRI enterography protocol after fractionally drinking 0.75 L of 2.5% mannitol solution within 1 h. Transverse T2-weighted turbo spin-echo (Half Fourier acquisition single-shot turbo spin-echo − HASTE) and the transverse T1-weighted gradient-echo (fast low angle shot − FLASH) sequences were analyzed in this study. Both sequences were acquired within the first 10 min of scanning before administering intravenous contrast in a fixed examination protocol. The field of view was adjusted to the individual patient’s size. Technical details of the MRI scanning parameters are listed in Table 2.

**Table 2:** Details of the scanning parameters

Scanner number	1		2		3	
Field strength	3 T		3 T		1.5 T	
Sequence	T1w GRE	T2w TSE	T1w GRE	T2w TSE	T1w GRE	T2w TSE
TR/TE (ms)	168/2.46	1,000/95	168/2.46	1,600/95	167/2.39	850/81
Flip angle (degree)	70	150	70	180	70	180
Slice thickness (mm)	5	5	5	5	6	6
Spacing between slices (mm)	0.5	0.5	0.5	0.5	0.6	0.6
Pixel spacing (typical range)	1.125/1.125	1.125/1.125	1.125/1.125	1.125/1.125	1.09375/ 1.09375	1.09375/ 1.09375
Acquisition matrix	320 × 158	320 × 194	320 × 210	320 × 194	320 × 203	256 × 167
Number of phase encoding steps	158	124	210	124	203	111
In plane phase encoding direction	Anterior–posterior					
Patient position	Head first					
Surface coil	Phased-array body coil					
Breathing regimen	Multi-breath-hold					

*GRE* Gradient-echo, *T1w* T1-weighted, *T2w* T2-weighted, *TE* Echo time, *TR* Repetition time, *TSE* Turbo spin-echo

### Segmentation

**Fig. 1:**
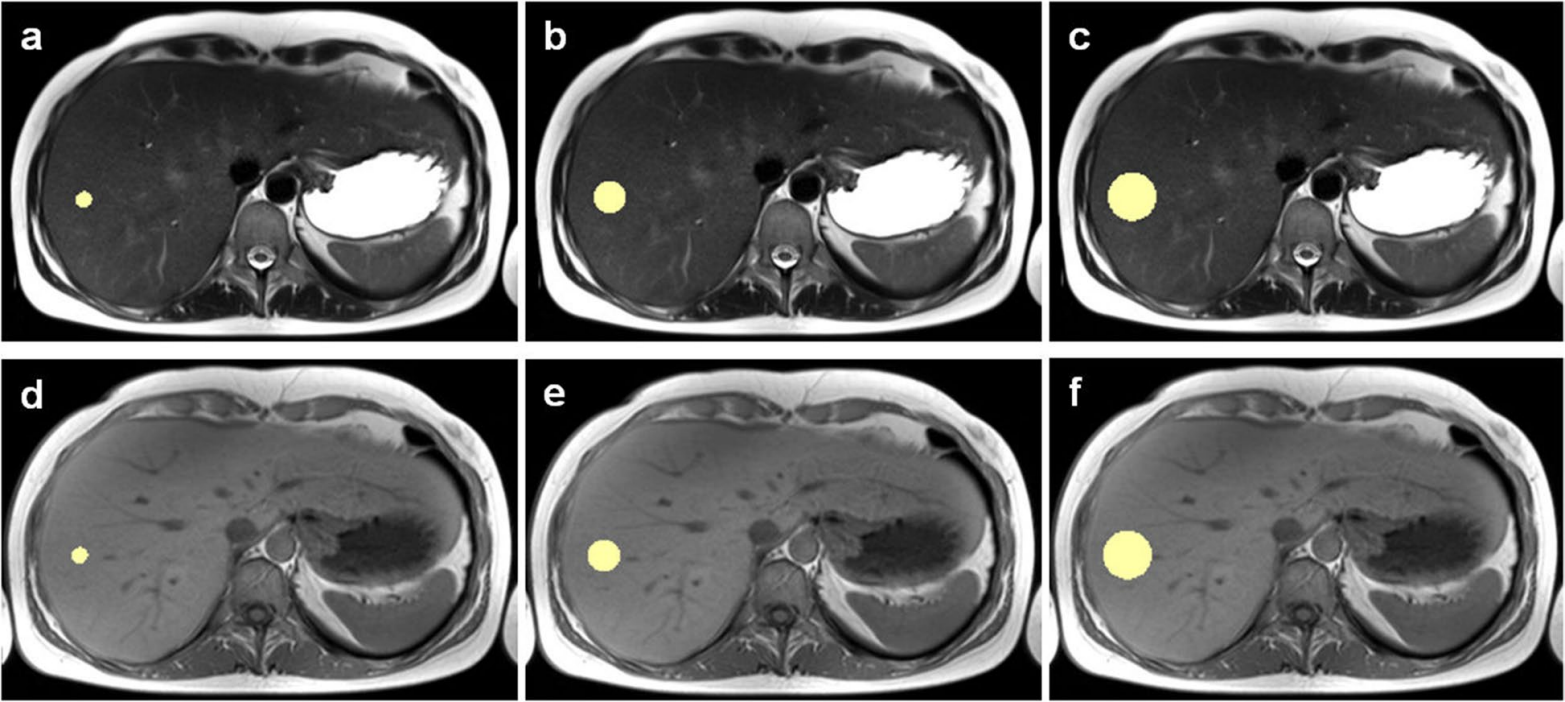
Volume of interest placement in the original images. Sphere-shaped volumes of interest of 10 mm (**a**, **d**), 20 mm (**b**, **e**), and 30 mm (**c**, **f**) diameter were placed in the right liver lobe attempting to exclude adjacent vessels. **a**–**c** T2-weighted turbo spin-echo sequences and (**d**–**f**) T1-weighted gradient-echo sequences, both of the same patient acquired on a 3-T scanner (scanner #2)

Sphere-shaped VOIs were drawn using 3D Slicer (Version 4.10.0, http://www.slicer.org) [22]. VOIs were placed in liver segments 5, 6, 7, or 8 by a radiologist with over 4 years of experience in MRI (L.J.J.), aiming to exclude large blood vessels. We chose the right lobe of the liver due to less motion artifacts from cardiac pulsations [23]. VOI diameters were set to 10, 20, and 30 mm since these are sizes to be expected for focal lesions such as metastases. Figure 1 shows an example of VOI placement in the original images.

### Computing parametric feature maps

**Fig. 2:**
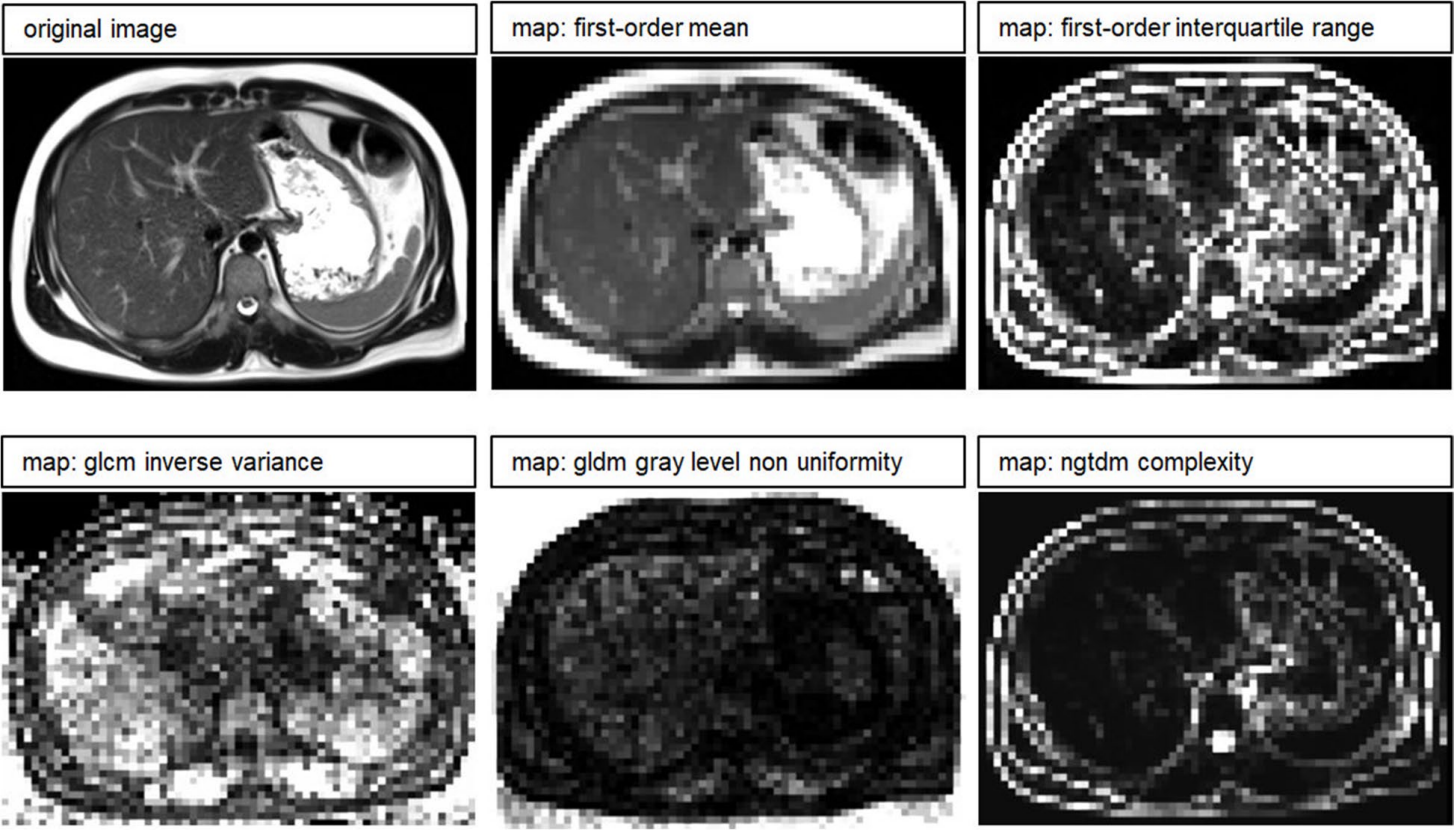
Exemplary feature maps. The original images were acquired on the 1.5-T scanner (scanner #3). The examples show the T2-weighted image and slices of the corresponding feature maps for first-order mean, first-order interquartile range, GLCM inverse variance, GLDM gray level non uniformity, and NGTDM complexity. Ninety-three features were included in our analysis, resulting in 93 parametric feature maps per patient for the T1-weighted and for the T2-weighted images

Parametric feature maps were computed using the pretested software tool of Kim et al. [21]. This tool can create parametric maps for any feature available in PyRadiomics [24]. Maps for 93 features were created per patient. Outsourced computing capacity accessible within the facility was used to shorten computation time. The voxel size and, therefore, the resolution of the maps was set to 5 mm for the computation since all chosen VOI diameters are multiples of this. With the *x*, *y*, and *z*-dimensions, the height and width of the voxels (which represent a grid of small VOIs) in the parametric maps can be defined, thus allowing the map's resolution to be adjusted. The *z*-resolution was adapted to match the slice thickness (5 mm). The *x*- and *y*-dimensions were set to 5 mm aiming for an adequate resolution of the images. The script containing the settings can be found in the Supplementary material (textfile S1). Figure 2 shows exemplary slices of different feature maps.

### Feature extraction from the original images and from the feature maps

All feature classes available in PyRadiomics (Version 3.0.1) except “shape features” were included [24]. Settings for the feature extraction were adjusted as recommended by the developers of PyRadiomics (see Supplementary material S2), and following the instructions of the Image Biomarker Standardization Initiative (see Supplementary material S3) [25]. Ninety-three features were extracted: 18 first-order features (energy, total energy, entropy, kurtosis, maximum, minimum, mean, median, interquartile range, skewness, range, mean absolute deviation, robust mean absolute deviation, root mean squared, variance, uniformity, 10th percentile, and 90th percentile) and 75 second- and higher-order features (24 gray level co-occurrence matrix − GLCM features, 14 gray level dependence matrix − GLDM features, 16 gray level run-length matrix − GLRLM features, 16 gray level size zone matrix − GLSZM features, and five neighboring gray tone difference matrix − NGTDM features) [24]. Shape features were not considered since VOI size was altered deliberately.

**Fig. 3:**
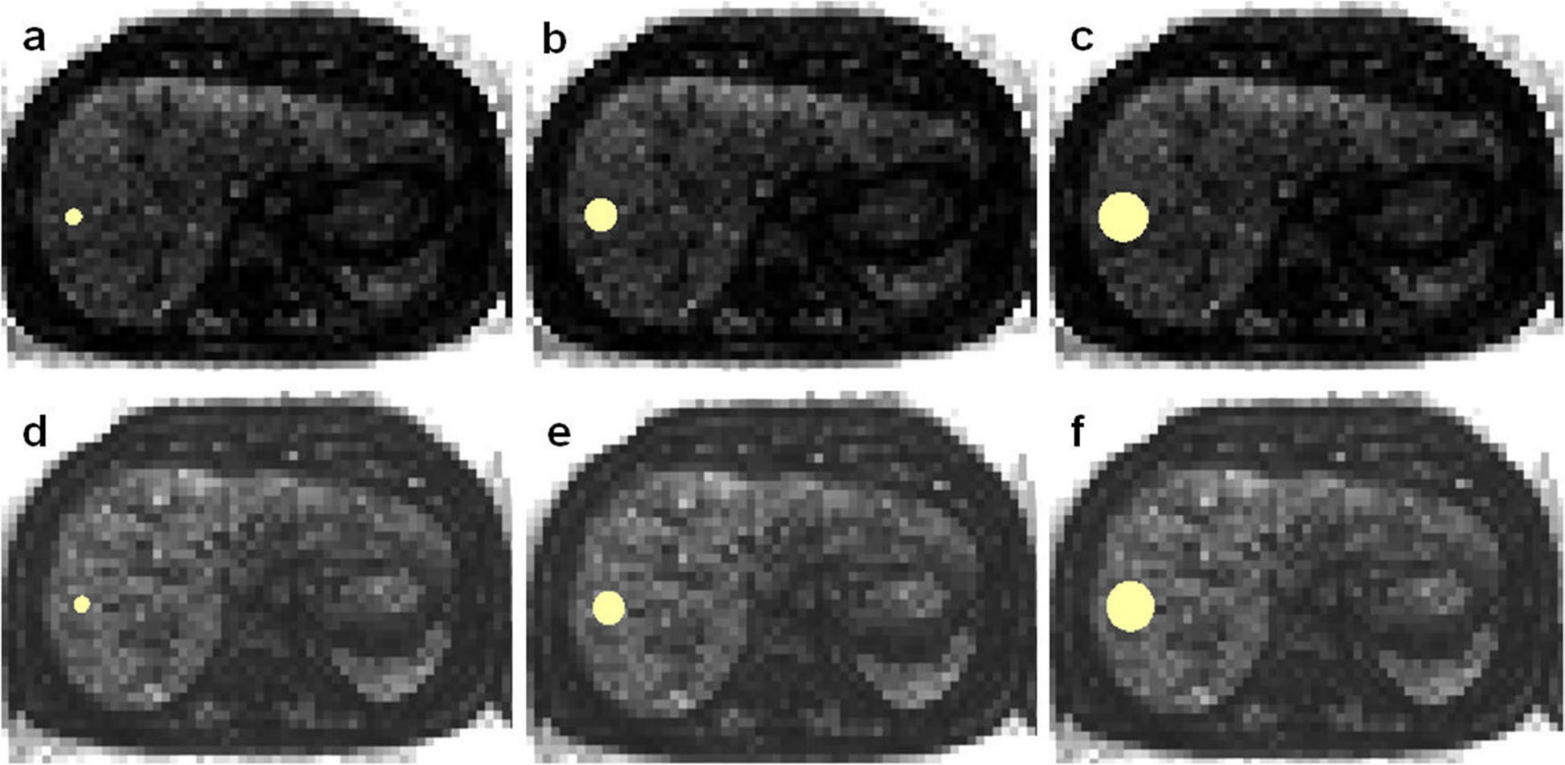
Volume of interest copy in the feature maps. Map of the feature first-order uniformity of the same patient as shown in Fig. 1. **a**–**c** Maps derived from T2-weighted images and (**d**–**f**) from the T1-weighted images. The volumes of interest (VOIs) of the conventional extraction were copied to the maps. The mean was extracted from the VOIs, resulting in the feature value for uniformity for the three different VOI sizes

For extraction from the parametric maps, the VOIs were copied from the original images into the maps of each patient. The mean of each VOI was directly retrieved and described the feature quantity of the respective map. Figure 3 shows the VOI placement in exemplary feature maps.

### Statistical analysis

Statistical analysis was performed using R (version 4.2.1, R Foundation for Statistical Computing) [26]. A *p* value < 0.05 was considered to indicate statistical significance. To assess the multivariable agreement, overall concordance correlation coefficients (OCCCs), according to Lin et al. [27] and Barnhart et al. [28], were calculated with the epiR package [29]. Like Rinaldi et al. [11], we considered features with an OCCC ≥ 0.85 stable and an OCCC < 0.85 nonreproducible. Reproducibility testing was applied to the results of the conventional feature extraction and the results of the parametric maps. Results were considered separately for each scanner.

## Results

### Conventional feature extraction from the original images

Across the VOI sizes 10, 20, and 30 mm, features with an OCCC ≥ 0.85 were limited to first-order features but without consistency across scanners. Only the features mean, median, root mean squared, and 10th percentile were stable across scanners and T1-weighted and T2-weighted sequences. Supplementary material S4 contains OCCC data of the conventional extraction for all features, sequences, and scanners. To provide the reader with an overview of grayscale behavior in the images, simple statistics for each case (minimum, maximum, range) are provided in Supplementary material 5.

Feature reproducibility increased when OCCCs were calculated without the smallest VOI of 10 mm diameter. In T1-weighted and T2-weighted sequences, also 90th percentile was stable across scanners. In T2-weighted images, three GLDM-features (glcm_Id, glcm_InverseVariance, glcm_Idm) also showed OCCCs ≥ 0.85. For both the 3-T scanners, 13 other features were reproducible in T1-weighted images but not in T2-weighted images. Also, on each scanner, other features were reproducible. Supplementary material S6 summarizes data for the OCCCs calculated without the 10 mm diameter VOIs. The results of the conventional extraction were already reported in a previous study [16].

### Feature extraction from the parametric maps

By directly extracting the feature quantities from the maps, OCCCs across the VOI sizes 10, 20, and 30 mm were above or equal to 0.85 for the same nine first-order features in T1-weighted and T2-weighted images. For both 3-T scanners, two second-/higher-order features (glrlm_RunLengthNonUniformity, glcm_JointEntropy) were reproducible in T1-weighted images. In addition, feature reproducibility increased overall, as shown in bar plots of the OCCCs in Supplementary material S7 separated per scanner for all VOI sizes. Supplementary material S8 contains numerical values of the OCCCs across the VOI sizes 10, 20, and 30 mm, and S9 across 20 and 30 mm. Although grayscale statistics can no longer be extracted from the maps, we also provide simple statistics for each case in supplementary material S10, where the minimum and maximum values were extracted from the corresponding map.

**Fig. 4:**
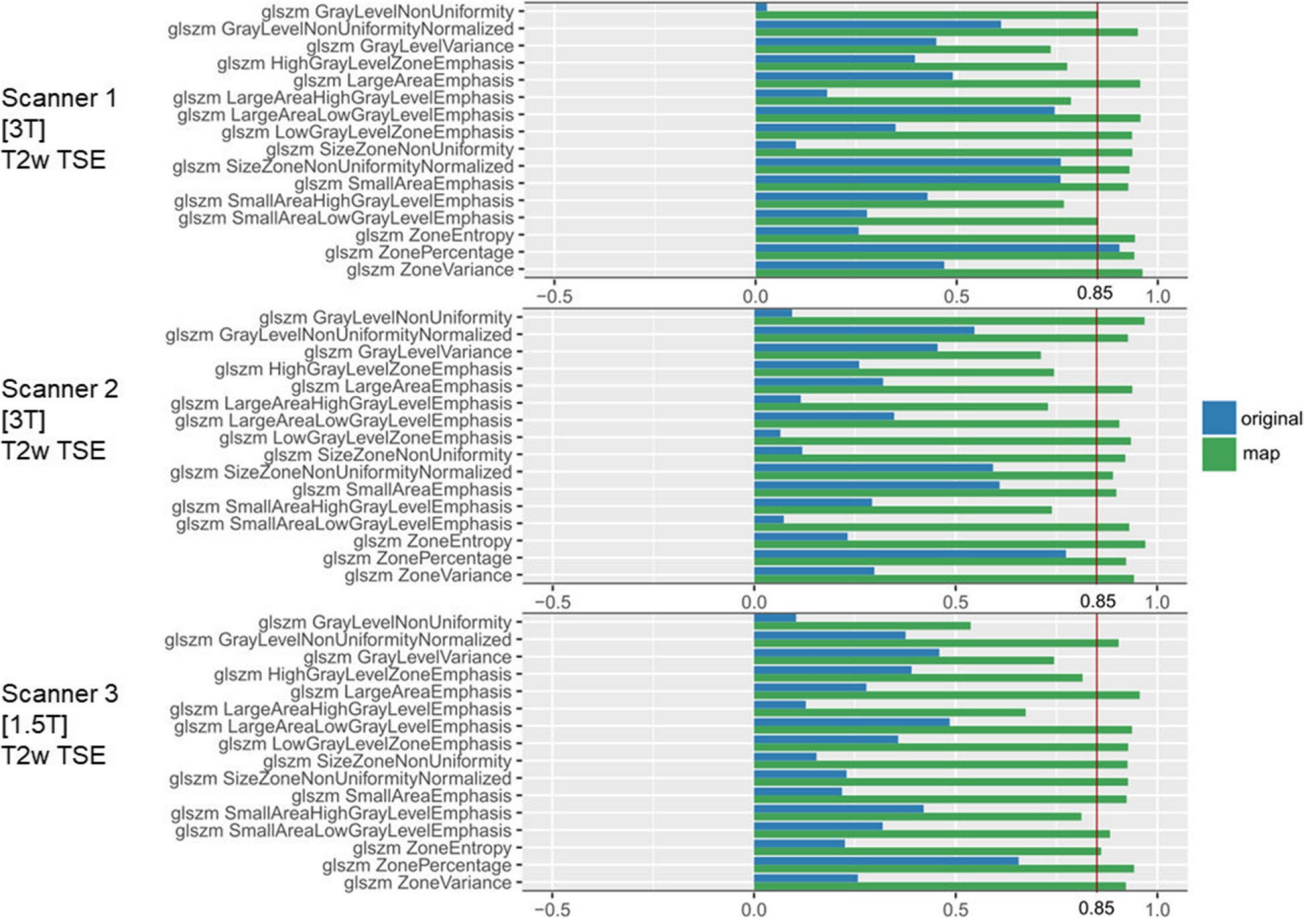
Overall concordance correlation coefficients (OCCCs) comparing GLSZM features from the original images and parametric maps. Bar plots of the OCCCs across volumes of interest (VOIs) with 20 and 30 mm diameter for each scanner. GLSZM features from T2-weighted images from the three scanners are shown with OCCC = 0.85 indicating feature reproducibility. The conventional extraction from the original image is shown in blue bars, and the map extraction in green bars. Feature reproducibility increased across the VOI sizes when features were extracted from the parametric maps

**Fig. 5:**
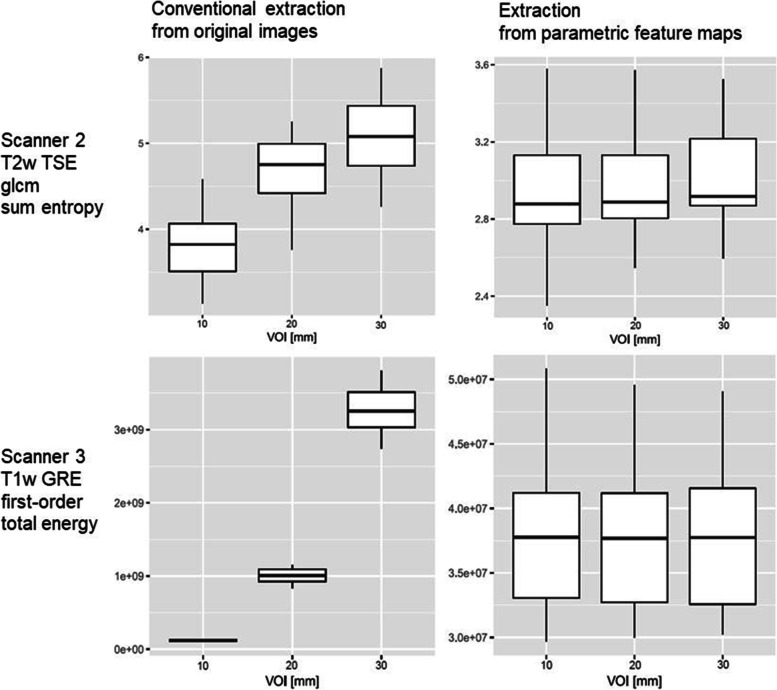
Boxplots of the conventional and the map extraction. Boxplots of the conventional and the map extraction for two examples are shown. In the map extraction, the boxplots are more congruous for the three volumes of interest (10, 20, and 30 mm) and the values are grouped closer around the mean

By excluding the smallest VOI from the OCCCs, stability improved in both T1-weighted and T2-weighted images. Nine first-order features were still reproducible in T1-weighted and 10 in T2-weighted images across all scanners. Seven additional second-/higher-order features became stable in T1-weighted, and 45 features in T2-weighted images. Agreement across the two 3-T scanners further increased with 39 additional reproducible features in T1-weighted and two additional features in T2-weighted images. OCCCs for the VOI sizes 20 and 30 mm separated per scanner are shown in Supplementary S11. Supplementary file S12 overviews the reproducible features for conventional and map extraction for the different scanners and sequences. Exemplary bar plots for OCCCs of 20 and 30 mm VOI sizes separated per scanner are shown in Fig. 4. In Fig. 5 boxplots of the conventional extraction and the map extraction of two examples across all three VOI sizes are compared.

### Reproducibility across scanners and scanning parameters

Overall, the consistency of reproducible features between the two 3-T scanners was higher than the 1.5-T scanner in the conventional and map extraction (as shown in Supplementary file S12). As shown in Table 2, scanning parameters like matrix, phase encoding steps, repetition time (TR), and flip angle differed on all three scanners. When comparing the T2-weighted images on the two 3-T scanners, TR and flip angle were higher on scanner 2. Of note, more T2-weighted features were reproducible in the map extraction on scanner 2 (see Supplementary file S12).

## Discussion

This study showed that the agreement of feature quantity across the VOI sizes improved when features were extracted from the parametric feature maps. When all VOI sizes were included, 4 out of the 93 features were reproducible in the conventional extraction and 9 in the map extraction across all sequences and scanners. When the smallest VOI size of 10 mm was excluded, reproducibility across the 20 and 30 mm VOIs showed a maximum of 18 features when extracted conventionally and a maximum of 57 features when sampled from the maps, with differences between T1-weighted or T2-weighted and field strength. Therefore, when the smallest VOI size was excluded, reproducibility increased in both the conventional extraction from the original images and particularly in the maps. Of note, agreement of features with OCCCs < 0.85 still improved in the map extraction (as shown in Fig. 4). Greater consistency of reproducible features existed across the two 3-T scanners, in contrast to the 1.5-T scanner. Even though feature reproducibility improved on all scanners, the individual feature behavior did not resemble across different scanning parameters.

In other words, converting MRI liver images to parametric feature maps before feature extraction increases the reproducibility of radiomics across different VOI sizes of nonpathological liver tissue. Many features gained stability (OCCCs ≥ 0.85), while others notably improved. Results were even better when the smallest VOI size (10 mm diameter) was excluded, possibly indicating that the maps perform better with increasing VOI size. The findings of our study make an essential contribution to the reproducibility and application of radiomics in clinical routine.

Other groups already discussed different volumes of interest as a possible constraint for reproducibility. Santinha et al. [30] proposed a volume threshold for radiomics in their MRI phantom study. They reported a loss of informative content when features were extracted from small VOIs, while their volumes ranged from 0.8 to 29.8 cm^3^. Further studies presented efforts to mitigate volume-confounding effects. Saltybaeva et al. [31] performed predictive modeling in a multicenter MRI study on glioblastoma multiforme. They used intra-class correlation coefficients to eliminate features correlating with tumor volume from their analysis. Fave et al. [32] detected volume-dependent features with Spearman correlation coefficients in their study on non-small cell lung cancer tumors with volumes ranging from 5 to 567 cm^3^. Aiming to correct the five features with strong volume correlation in their study, they divided the values by the total number of voxels in the region of interest. Roy et al. [17] investigated the correlation between tumor volume and features in breast cancer lesions on MRI. In their approach, features with linear correlations were divided by the tumor volume, and inversely proportional features were multiplied. Features with nonlinear correlations were processed with principal component analysis, but some features were still volume-dependent even after dimension reduction. Shafiq-ul-Hassan et al. [33, 34] improved the reproducibility of radiomics by normalization of voxel size. Two other groups resampled the VOIs to isometric voxels before the feature extraction [35, 36]. In other studies, features prone to different volumes were excluded stepwise from the applied feature set [13, 37, 38]. In the present study, the calculation of parametric maps before feature extraction renders mathematical corrections of volume dependencies unnecessary. As a side effect, parametric maps also allow visualization of feature behavior.

Improving volume independence for liver-derived features could have been valuable to other study designs. For example, Zhang et al. [39] attempted to predict therapy response in patients with colorectal liver metastases. They delineated free-hand regions of interest around the largest cross-sectional area of the liver lesions in T2-weighted images acquired on a 3-T scanner. Lesions with a diameter greater than 1 cm were selected. They extracted five features (mean, variance, skewness, kurtosis, and entropy). Calculating parametric feature maps might have been beneficial in their study since variance, skewness, kurtosis, and entropy showed increased reproducibility across VOI sizes when extracted from the feature maps (yet, mean is also reproducible when derived conventionally from the original images). Another group [40] analyzed links between textural features and microvascular invasion in hepatocellular carcinoma in post-contrast-enhanced T1-weighted images. Resected specimens served as the reference standard. Features were also extracted based on the largest cross-sectional area of each lesion. Increasing the feature reproducibility by preprocessing images to parametric maps might also have been helpful, despite the influence of contrast media on the maps would still remain unclear.

Although by the map extraction, feature reproducibility improved throughout all three included scanners (two 3-T scanners and one 1.5-T scanner) scanner-wise, it is worth mentioning that the behavior of the individual features did not resemble across field strengths and scanning parameters. Different scanning parameters were applied on each scanner (*e.g.,* phase encoding steps, matrix, TR, and flip angle), as shown in Table 2. Of note, in the map extraction, most features were reproducible on scanner #2 (one of the 3-T scanners, as indicated in Table 2), holding the highest flip angle and TR on T2-weighted sequences, conceivably indicating an enhancement of feature reproducibility through a better signal-to-noise ratio [41]. The influence of different scanners and vendors, field strength, and scanning parameters are known obstacles concerning the reproducibility of MRI-derived radiomics and cannot be bypassed by the parametric feature maps [17, 42–44].

Our study has some limitations. Verification of the results in a larger patient cohort would have been desirable. Since enrollment was conducted strictly and only patients without liver lesions and abnormal signal alterations to the liver parenchyma were included, only 66 patients were eligible for a long retrospective screening period. Repeating the analysis in other organs would also have been interesting, but the spacious variation of the VOI would have been challenging in smaller organs like the spleen or pancreas. The limitation of the parametric feature maps is the required computing power. Calculating one feature map stack requires several hours of computing time. The computation process might have been accelerated if the original image stack had been cropped, which could possess a future applicability mode. Strictly limiting the map to the VOI, however, would lead to errors along the edges. But cropping the original image to a few voxels close to the VOI and then extracting the mean from the VOI in the map would reduce the required computing power and enhance effectiveness. Therefore, implementing a cropping step seems inevitable in further applications. It might be seen as a drawback that VOIs were drawn manually in our study. Since we aimed for control and transparency of the results, we preferred manual segmentation.

Software-based conversion of images into parametric feature maps before feature extraction improves feature reproducibility across different VOI sizes in normal liver tissue. Since the graphical presentation of the features in the maps provides insights into their behavior, disturbing factors such as artifacts may be elucidated. The general applicability of parametric maps to radiological images could also enable correction for differently sized VOIs in further studies on radiomics. Testing the applicability of the feature maps on focal liver lesions would be a future perspective.

### Supplementary Information

**Additional file 1.** Script of the parametric maps.

**Additional file 2.** PyRadiomics settings for the conventional extraction.

**Additional file 3.** IBSI reporting guidelines.

**Additional file 4.** OCCC data for the VOI sizes of 10, 20, and 30 mm from the original images (conventional extraction).

**Additional file 5.** Overview of grayscale behavior of the original images.

**Additional file 6.** OCCC data for the VOI sizes of 20 and 30 mm from the original images (conventional extraction).

**Additional file 7.** Barplots of the OCCCs per scanner (VOI sizes of 10, 20, and 30 mm).

**Additional file 8. **OCCC data for the VOI sizes of 10, 20, and 30 mm from the maps (map extraction).

**Additional file 9.** OCCC data for the VOI sizes of 20 and 30 mm from the maps (map extraction).

**Additional file 10. **Overview of grayscale behavior of the feature maps.

**Additional file 11.** Barplots of the OCCCs per scanner (VOI sizes of 20 and 30 mm).

**Additional file 12.** Overview of the reproducible features.

## Data Availability

The datasets used and/or analyzed during the current study are available from the corresponding author on reasonable request.

## Abbreviations

MRI: Magnetic resonance imaging
OCCC: Overall concordance correlation coefficient
TR: Repetition time
VOI: Volume of interest
