# Supplementary material for: The role of parametric feature maps to correct different volume of interest sizes: an in vivo liver MRI study
Source: Eur Radiol Exp. 2023 Sep 6;7:48. doi: 10.1186/s41747-023-00362-9 (PMC10480134; doi:10.1186/s41747-023-00362-9)
Supplement: Supplementary file 1 — Additional file 1. Script of the parametric maps. [file 41747_2023_362_MOESM1_ESM.pdf]

```
# coding=utf-8

###############
#DEPENDENCIES:#
###############

!pip install pynrrd
!pip install SimpleITK
!pip install pyradiomics

from __future__ import print_function # für nrrd to dicom
import sys, time # für nrrd to dicom

import numpy as np
import nrrd
import os
import csv
import SimpleITK as sitk
import six
import math
import pandas as pd

import multiprocessing
from joblib import Parallel, delayed

from radiomics import featureextractor
```

```
from google.colab import drive



###########
#SETTINGS:#
###########

#number of jobs for multiprocessing, -1 should use all cpus
num_jobs = -1

#where is the DICOM folder, mount GoogleDrive at /content/gdrive/
drive.mount("/content/gdrive")
basefolder = "/content/gdrive/MyDrive/RFE_1/"
DICOMfolder = basefolder+"DICOM/"

#Extraction Settings: mit ""
YAMLfile = basefolder+"params.yaml"
target_resolution_unit = "mm" #options: px or mm

target_resolution_x = 5
target_resolution_y = 5
target_resolution_z = 5
```

```
#############
#############
#############
# image, voxel_size_x, voxel_size_y and voxel_size_z are needed globally througot the program




#############
#LOAD DICOM:#
#############



#read DICOM folder as image
reader = sitk.ImageSeriesReader()
dicom_names = reader.GetGDCMSeriesFileNames(DICOMfolder)
reader.SetFileNames(dicom_names)
image = reader.Execute()

#debug - get image size and pixel spacing
dicom_image_size = image.GetSize()
dicom_pixel_spacing = image.GetSpacing()

#debug
print("Image Size X [px]:", dicom_image_size[0])
print("Image Size Y [px]:", dicom_image_size[1])
print("Image Size Z [slices]:", dicom_image_size[2])
```

```
print("Pixel Spacing X [mm/px]:", dicom_pixel_spacing[0])

print("Pixel Spacing Y [mm/px]:", dicom_pixel_spacing[1])

print("Pixel Spacing Z [mm/slice]:", dicom_pixel_spacing[2])




##################################

#CALCULATE SINGLE VOXEL DIMENSION#

##################################


#get image size and pixel spacing

dicom_image_size = image.GetSize()

dicom_pixel_spacing = image.GetSpacing()


#if target resulution is set to pixel, then voxel dimension euqals

if target_resolution_unit == "px":

    voxel_size_x = target_resolution_x

    voxel_size_y = target_resolution_y

    voxel_size_z = target_resolution_z


#if target resulution is set to millimeter, then voxel dimension needs to be calculated, round to nearest full pixel

elif target_resolution_unit == "mm":

    voxel_size_x = round(target_resolution_x/dicom_pixel_spacing[0])
```

```
    voxel_size_y = round(target_resolution_y/dicom_pixel_spacing[1])

    voxel_size_z = round(target_resolution_z/dicom_pixel_spacing[2])


#debug

print("Voxel Size X [px]:", voxel_size_x, "| Voxel Size X [mm]:", voxel_size_x*dicom_pixel_spacing[0])

print("Voxel Size Y [px]:", voxel_size_y, "| Voxel Size Y [mm]:", voxel_size_y*dicom_pixel_spacing[1])

print("Voxel Size Z [slices]:", voxel_size_z, "| Voxel Size Z [mm]:", voxel_size_z*dicom_pixel_spacing[2])




######################

#FUNC: SELECT SLICES#

######################


#returns a block of slices of the targeted size in z direction, i.e. the z voxel size

#needs the original image that is to be sliced, the targeted size in z direction

#and the index of the slice to be returned, i.e. the n-th set of slices while iterating through the dataset

#important: everything starts with index 0 and the last slice is not included, examples: slicing 0:4 return slices 0,1,2,3; 4:8 returns 4,5,6,7


def select_z_block(image,voxel_size_z,z_step):


    #debug

    #print("Select slices from", z_step*voxel_size_z, "to", z_step*voxel_size_z+voxel_size_z-1)

    #from image get all pixels in x and y direction, and slices for the slice_index (the ":,:," at the beginning stands for all in x and y dimension, otherwise could be specified, e.g. 0:dicom_image_size_x)
```

```
    z_block = image[:,:,z_step*voxel_size_z:z_step*voxel_size_z+voxel_size_z]

    #debug
    #writer = sitk.ImageFileWriter()
    #writer.SetFileName(basefolder+"test_block"+str(slice_index)+".nrrd")
    #writer.Execute(slice_block)

    return (z_block)


#debug - test run select_slices
#test_block = select_slices(image,voxel_size_z,0)

#FUNC END: SELECT SLICES FUNCTION





########################
#FUNC: CREATE GRID MASK#
########################

#returns a grid of labels when a block of slices is parsed
#needs a block of slices and targeted size in y and y direction, i.e. the x and y voxel size
```

```
def create_x_y_mask(z_block,starting_label,voxel_size_x,voxel_size_y):


    #debug

    #print("Creating grid of", voxel_size_x, "x", voxel_size_y, "pixels")

    z_block_size = z_block.GetSize()


    #math.ceil rounds up to nearest integer

    x_steps = math.ceil(z_block_size[0]/voxel_size_x)

    y_steps = math.ceil(z_block_size[1]/voxel_size_y)

    label = starting_label


    #use z_block as x_y_mask (keeps alles spacings and directions)

    #simply use all full dimensions ("[:,:,:]"), but if just x_y_mask = z_block, z_block get overwritten...

    x_y_mask = z_block[:,:,:]


    #increase bit size of x_y_mask (can hold up to 4,294,967,295 labels)

    x_y_mask = sitk.Cast(x_y_mask,sitk.sitkUInt32)


    for x_step in range(0,x_steps):

        for y_step in range(0,y_steps):

x_y_mask[x_step*voxel_size_x:x_step*voxel_size_x+voxel_size_x,y_step*voxel_size_y:y_step*voxel_siz
e_y+voxel_size_y,:] = label

            label = label+1

                #END loop y_step

    #END loop x_step
```

```
    #debug
    #writer = sitk.ImageFileWriter()
    #writer.SetFileName(basefolder+"test_block"+str(label)+"-label.nrrd")
    #writer.Execute(x_y_mask)

    return (x_y_mask,label)

#debug - test run create gridmask
#test_grid = create_x_y_mask(image,1,voxel_size_x,voxel_size_y)

#FUNC END: CREATE GRID MASK




########################
#FUNC: EXTRACT FEATURES#
########################

#returns a dictionary filled with the features value per label
#needs the image for which the features should be calculated
#calls the functions "select_z_block" and "creat_y_x_mask"


def run_pyrad(label,z_block,x_y_mask):
```

```
    #instantiate the feature extractor with the YAMLfile
    extractor = featureextractor.RadiomicsFeatureExtractor(YAMLfile)

    #dictionary to collect results
    z_step_results = []

    #run extraction
    z_step_results.append(extractor.execute(z_block,x_y_mask,label))

    return(z_step_results)


def extract_features(image,voxel_size_x,voxel_size_y,voxel_size_z):


    #####################
    #ITERATE OVER BLOCKS#
    #####################

    #collect results (dictionary)
    extraction_results = []

    #get image size
    image_size = image.GetSize()
```

```
#get number of steps in z direction, i.e. the number of z_blocks
z_steps = math.ceil(image_size[2]/voxel_size_z)

#get labels per z_step
x_steps = math.ceil(image_size[0]/voxel_size_x)
y_steps = math.ceil(image_size[1]/voxel_size_y)

total_steps = z_steps * x_steps * y_steps

#apparently max. 32767 labels possible, when data type Int32
block_start_label = 1

for z_step in range(0,z_steps):

    #print z_step progress
    print("Calculating",z_step+1,"of",z_steps,"total z_steps")

    #get z_block for z_step
    z_block = select_z_block(image,voxel_size_z,z_step)

    #get x_y_grid for z_step
    x_y_mask,block_end_label = create_x_y_mask(z_block,block_start_label,voxel_size_x,voxel_size_y)

    #debug
    #print(x_y_mask.GetPixelIDTypeAsString())
```

```
        #debug
        #writer = sitk.ImageFileWriter()
        #writer.SetFileName(basefolder+"z_block"+str(z_step)+".nrrd")
        #writer.Execute(z_block)

        #debug
        #writer = sitk.ImageFileWriter()
        #writer.SetFileName(basefolder+"z_block"+str(z_step)+"-label.nrrd")
        #writer.Execute(x_y_mask)

        #n_jobs -1 should activate all cpus available
        if __name__ == "__main__":
            z_step_results = Parallel(n_jobs=num_jobs)(delayed(run_pyrad)(label,z_block,x_y_mask) for label
in range(block_start_label,block_end_label))

        #collect z_step_results
        extraction_results.extend(z_step_results)

        #define start label for next z_step
        block_start_label = block_end_label

    #convert results to pandas dataframe (way easier handling, like r dataframe)
    #no idea how this actually works, got it from here:
https://stackoverflow.com/questions/52902158/how-to-create-a-pandas-dataframe-from-a-list-of-
ordereddicts
    extraction_results = pd.DataFrame([extraction_results[i][0] for i, j in enumerate(extraction_results)])
```

```
    return(extraction_results)

#FUNC END: EXTRACT FEATURES

#run extract features
extraction_results = extract_features(image,voxel_size_x,voxel_size_y,voxel_size_z)

##################
#FUNC: CREATE MAP#
##################

#this function creates a map for a given feature

def
create_map(image,extraction_results,feature_column_index,voxel_size_x,voxel_size_y,voxel_size_z):

    #get number of steps in z direction, i.e. the number of z_blocks
    image_size = image.GetSize()

    x_steps = math.ceil(image_size[0]/voxel_size_x)
    y_steps = math.ceil(image_size[1]/voxel_size_y)
    z_steps = math.ceil(image_size[2]/voxel_size_z)
```

```
    #set starting label
    label = 1

    #define new map from image
    feature_map = image[:,:,:]

    #increase bit size of map to floating point
    feature_map = sitk.Cast(feature_map,sitk.sitkFloat32)

    for z_step in range(0,z_steps):
        for x_step in range(0,x_steps):
            for y_step in range(0,y_steps):
                #.item() is important to get the actual integer out of the cell (way back at the end of the
following line)

feature_map[x_step*voxel_size_x:x_step*voxel_size_x+voxel_size_x,y_step*voxel_size_y:y_step*voxel_
size_y+voxel_size_y,z_step*voxel_size_z:z_step*voxel_size_z+voxel_size_z] =
extraction_results.iat[label-1,feature_column_index].item()
                label = label+1
            #END loop y_step
        #END loop x_step
    #END loop z_step

    return(feature_map)

#test run create map
```

```
#create_map(image,extraction_results,22,voxel_size_x,voxel_size_y,voxel_size_z)

#FUNC END: CREATE MAP




######################

#FUNC: WRITE MAPS NRRD#

######################


#this is the function that acutally writes out the maps as files.

#needs the image for which the features were calculated, the extraction_results,

#as well as the resolution of the maps (voxel_size_x,voxel_size_y,voxel_size_z)

#calls "create map" for every feature found in extraction_results



def write_maps(image,extraction_results,voxel_size_x,voxel_size_y,voxel_size_z):



    ######################

    #ITERATE OVER RESULTS#

    ######################


    #get index of first feature in dictionorary

    #this is the feature after "diagnostics_Mask-original_CenterOfMass"
```

```
    first_feature_column_index = extraction_results.columns.get_loc("diagnostics_Mask-
original_CenterOfMass")+1

    #the last column contains the last feature

    last_feature_column_index = len(extraction_results.columns)



    #iterate over all features in results:

    for feature_column_index in range(first_feature_column_index,last_feature_column_index):

        feature_map =
create_map(image,extraction_results,feature_column_index,voxel_size_x,voxel_size_y,voxel_size_z)


        #debug print column name

        #print(extraction_results.columns[feature_column_index])


        #debug write nrrd of map

        writer = sitk.ImageFileWriter()

        writer.SetFileName(basefolder+extraction_results.columns[feature_column_index]+".nrrd")

        writer.Execute(feature_map)



    #debug write csv file with all results

    #with open(basefolder+"extraction_results.csv","w") as foutput:

    #    w = csv.writer(foutput)

    #    #get the column names using the key

    #    w.writerow(extraction_results[0].keys())

    #

    #    #now write the results using the values for every entry in the dictionary
```

```
    #    #get length of dictionary with "len"
    #    for i in range (0,len(extraction_results)):
    #        w.writerow(extraction_results[i].values())



#test run create maps
write_maps(image,extraction_results,voxel_size_x,voxel_size_y,voxel_size_z)


#FUNC END: WRITE MAPS
```
