# Supplementary material for: The role of parametric feature maps to correct different volume of interest sizes: an in vivo liver MRI study
Source: Eur Radiol Exp. 2023 Sep 6;7:48. doi: 10.1186/s41747-023-00362-9 (PMC10480134; doi:10.1186/s41747-023-00362-9)
Supplement: Supplementary file 2 — Additional file 2. PyRadiomics settings for the conventional extraction. [file 41747_2023_362_MOESM2_ESM.pdf]

```yaml
imageType:
  Original: {}

featureClass:
    firstorder:
    glcm:
    glrlm:
    glszm:
    gldm:
    ngtdm:

setting:
  binWidth: 5
  voxelArrayShift: 300
  correctMask: true
```
