# Supplementary material for: The role of parametric feature maps to correct different volume of interest sizes: an in vivo liver MRI study
Source: Eur Radiol Exp. 2023 Sep 6;7:48. doi: 10.1186/s41747-023-00362-9 (PMC10480134; doi:10.1186/s41747-023-00362-9)
Supplement: Supplementary file 3 — Additional file 3. IBSI reporting guidelines. [file 41747_2023_362_MOESM3_ESM.pdf]

IBSI guidelines for reporting on radiomics studies

Checklist - Version 1.0 (October 2019)

This checklist focuses specifically on in-depth reporting of studies involving radiomics. Other reporting guidelines may be applicable as well, e.g. STROBE (observational studies), CONSORT (randomised trials).

Not all items may be applicable. Indicate only applicable items.

**Topic**		**Item**	**Description**	**Page** Please note: We refer to sections and paragraphs as the page number may not be persistent.
**Patient**				
Region of interest[1]		1	Describe the region of interest that is being imaged.	M&M, *Study Population and definition of target lesion*
Patient preparation		2a	Describe specific instructions given to patients prior to image acquisition, e.g. fasting prior to imaging.	M&M, *Image acquisition*
		2b	Describe administration of drugs to the patient prior to image acquisition, e.g. muscle relaxants.	M&M, *Image acquisition*
		2c	Describe the use of specific equipment for patient comfort during scanning, e.g. ear plugs.	No special measures
Radioactive tracer	PET, SPECT	3a	Describe which radioactive tracer was administered to the patient, e.g. 18F-FDG.	3a to e: not applicable
	PET, SPECT	3b	Describe the administration method.	
	PET, SPECT	3c	Describe the injected activity of the radioactive tracer at administration.	
	PET, SPECT	3d	Describe the uptake time prior to image acquisition.	
	PET, SPECT	3e	Describe how competing substance levels were controlled.[2]	
Contrast agent		4a	Describe which contrast agent was administered to the patient.	4a to e: No contrast enhanced images considered
		4b	Describe the administration method.	
		4c	Describe the injected quantity of contrast agent.	
		4d	Describe the uptake time prior to image acquisition.	
		4e	Describe how competing substance levels were controlled.	
Comorbidities		5	Describe if the patients have comorbidities that affect imaging.[3]	M&M, *Study population*

[1] Also referred to as volume of interest.

[2] An example is glucose present in the blood which competes with the uptake of 18F-FDG tracer in tumour tissue. To reduce competition with the tracer, patients are usually asked to fast for several hours and a blood glucose measurement may be conducted prior to tracer administration.

[3] An example of a comorbidity that may affect image quality in 18F-FDG PET scans are type I and type II diabetes melitus, as well as kidney failure.

Acquisition[4]

Acquisition protocol		6	Describe whether a standard imaging protocol was used, and where its description may be found.	
Scanner type		7	Describe the scanner type(s) and vendor(s) used in the study.	6 to 9a: M&M, *Image acquisition*
Imaging modality		8	Clearly state the imaging modality that was used in the study, e.g. CT, MRI.	
Static/dynamic scans		9a	State if the scans were static or dynamic.	
	Dynamic scans	9b	Describe the acquisition time per time frame.	9b and c: Scans were static
	Dynamic scans	9c	Describe any temporal modelling technique that was used.	
Scanner calibration		10	Describe how and when the scanner was calibrated.	6 to 10: M&M, *Image acquisition*
Patient instructions		11	Describe specific instructions given to the patient during acquisition, e.g. breath holding.	No specific instructions
Anatomical motion correction		12	Describe the method used to minimise the effect of anatomical motion.	No specific methods
Scan duration		13	Describe the duration of the complete scan or the time per bed position.	13 M&M, *Image acquisition* and table 2<br>14 1o 16: not applicable
Tube voltage	CT	14	Describe the peak kilo voltage output of the X-ray source.	
Tube current	CT	15	Describe the tube current in mA.	
Time-of-flight	PET	16	State if scanner time-of-flight capabilities are used during acquisition.	
RF coil	MRI	17	Describe what kind RF coil used for acquisition, incl. vendor.	
Scanning sequence	MRI	18a	Describe which scanning sequence was acquired.	17 to 21: M&M, *Image acquisition* and table 2
	MRI	18b	Describe which sequence variant was acquired.	
	MRI	18c	Describe which scan options apply to the current sequence, e.g. flow compensation, cardiac gating.	
Repetition time	MRI	19	Describe the time in ms between subsequent pulse sequences.	22 not applicable
Echo time	MRI	20	Describe the echo time in ms.	23 and 24: M&M, *Image acquisition* and table 2
Echo train length	MRI	21	Describe the number of lines in k-space that are acquired per excitation pulse.	
Inversion time	MRI	22	Describe the time in ms between the middle of the inverting RF pulse to the middle of the excitation pulse.	25 not applicable
Flip angle	MRI	23	Describe the flip angle produced by the RF pulses.	
Acquisition type	MRI	24	Describe the acquisition type of the MRI scan, e.g. 3D.	26 and 27: M&M, *Image acquisition* and table 2
k-space traversal	MRI	25	Describe the acquisition trajectory of the k-space.	
Number of averages/ excitations	MRI	26	Describe the number of times each point in k-space is sampled.	
Magnetic field strength	MRI	27	Describe the nominal strength of the MR magnetic field.	

Reconstruction[5]

In-plane resolution		28	Describe the distance between pixels, or alternatively the field of view and matrix size.	
Image slice thickness		29	Describe the slice thickness.	28 to 30: M&M, *Image acquisition* and table 2<br>31a to 31 c: not applicable
Image slice spacing		30	Describe the distance between image slices.[6]	
Convolution kernel	CT	31a	Describe the convolution kernel used to reconstruct the image.	
	CT	31b	Describe settings pertaining to iterative reconstruction algorithms.	
Exposure	CT	31c	Describe the exposure (in mAs) in slices containing the	

[4] Many acquisition parameters may be extracted from DICOM header meta-data, or calculated from them.
[5] Many reconstruction parameters may be extracted from DICOM header meta-data.
[6] Spacing between image slicing is commonly, but not necessarily, the same as the slice thickness,.

			region of interest.	
Reconstruction method	PET	32a	Describe which reconstruction method was used, e.g. 3D OSEM.	
	PET	32b	Describe the number of iterations for iterative reconstruction.	
	PET	32c	Describe the number of subsets for iterative reconstruction.	32a to 34a: not applicable
Point spread function modelling	PET	33	Describe if and how point-spread function modelling was performed.	
Image corrections	PET	34a	Describe if and how attenuation correction was performed.	
	PET	34b	Describe if and how other forms of correction were performed, e.g. scatter correction, randoms correction, dead time correction etc.	
Reconstruction method	MRI	35a	Describe the reconstruction method used to reconstruct the image from the k-space information.	35a to 35b: M&M, *Image acquisition* and table 2
	MRI	35b	Describe any artifact suppression methods used during reconstruction to suppress artifacts due to undersampling of k-space.	
Diffusion-weighted imaging	DWI-MRI	36	Describe the b-values used for diffusion-weighting.	36: not applicable
**Image registration**				
Registration method		37	Describe the method used to register multi-modality imaging.	not applicable
**Image processing - data conversion**				
SUV normalisation	PET	38	Describe which standardised uptake value (SUV) normalisation method is used.	not applicable
ADC computation	DWI-MRI	39	Describe how apparent diffusion coefficient (ADC) values were calculated.	not applicable
Other data conversions		40	Describe any other conversions that are performed to generate e.g. perfusion maps.	not applicable
**Image processing - post-acquisition processing**				
Anti-aliasing		41	Describe the method used to deal with anti-aliasing when down-sampling during interpolation.	41 to 42: M&M, Study population and *Image acquisition*
Noise suppression		42	Describe methods used to suppress image noise.	
Post-reconstruction smoothing filter	PET	43	Describe the width of the Gaussian filter (FWHM) to spatially smooth intensities.	
Skull stripping	MRI (brain)	44	Describe method used to perform skull stripping.	not applicable
Non-uniformity correction[7]	MRI	45	Describe the method and settings used to perform non-uniformity correction.	not applicable
Intensity normalisation		46	Describe the method and settings used to normalize intensity distributions within a patient or patient cohort.	No normalization was performed
Other post-acquisition processing methods		47	Describe any other methods that were used to process the image and are not mentioned separately in this list.	not applicable
**Segmentation**				
Segmentation method		48a	Describe how regions of interest were segmented, e.g. manually.	
		48b	Describe the number of experts, their expertise and consensus strategies for manual delineation.	48a to d: M&M, *Image analysis*
		48c	Describe methods and settings used for semi-automatic and fully automatic segmentation.	
		48d	Describe which image was used to define segmentation in case of multi-modality imaging.	
Conversion to mask		49	Describe the method used to convert polygonal or mesh-based segmentations to a voxel-based mask.	not applicable
**Image processing - image interpolation**				

[7] Also known as bias-field correction.

Interpolation method		50a	Describe which interpolation algorithm was used to interpolate the image.	
		50b	Describe how the position of the interpolation grid was defined, e.g. align by center.	
		50c	Describe how the dimensions of the interpolation grid were defined, e.g. rounded to nearest integer.	No specific image interpolation applied
		50d	Describe how extrapolation beyond the original image was handled.	
Voxel dimensions		51	Describe the size of the interpolated voxels.	
Intensity rounding	CT	52	Describe how fractional Hounsfield Units are rounded to integer values after interpolation.	
**Image processing - ROI interpolation**				
Interpolation method		53	Describe which interpolation algorithm was used to interpolate the region of interest mask.	No specific image interpolation applied
Partially masked voxels		54	Describe how partially masked voxels after interpolation are handled.	
**Image processing - re-segmentation**				
Re-segmentation methods		55	Describe which methods and settings are used to re-segment the ROI intensity mask.	No re-segmentation was performed.
**Image processing - discretisation**				
Discretisation method[8]		56a	Describe the method used to discretise image intensities.	Fixed bin size provided in configuration file. No further steps to discretize intensities.
		56b	Describe the number of bins (FBN) or the bin size (FBS) used for discretisation.	
		56c	Describe the lowest intensity in the first bin for FBS discretisation.[9]	0 (voxelshift of 1000 applied, see also PyRadiomics configuration)
**Image processing - image transformation**				
Image filter[10]		57	Describe the methods and settings used to filter images, e.g. Laplacian-of-Gaussian.	No filters applied
**Radiomics feature computation**				
Feature set		58	Describe which set of radiomics features is computed and refer to their definitions or provide these.	58 to 61: M&M,*Radiomic Feature Extraction*
IBSI compliance		59	State if the software used to extract the set of features is able to reproduce the IBSI feature reference values.[11]	
Robustness		60	Describe how robustness of the features was assessed, e.g. test-retest analysis.	
Software availability		61	Describe which software and version was used to compute features.	
**Radiomics feature computation - texture parameters**				
Texture matrix aggregation		62	Define how texture-matrix based features were computed from underlying texture matrices.	62 to 71: We can only refer to the source code of PyRadiomics here
Distance weighting		63	Define how CM, RLM, NGTDM and NGLDM weight distances, e.g. no weighting.	
CM symmetry		64	Define whether symmetric or asymmetric co-occurrence	

[8] Discretisation may be performed separately to create intensity-volume histograms. If this is indeed the case, this should be described as well.

[9] This is typically set by range re-segmentation.

[10] The IBSI has not introduced image transformation into the standardised image processing scheme, and is in the process of benchmarking various common filters. This section may therefore be expanded in the future.

[11] A software is compliant if and only if it is able to reproduce the feature reference values for the digital phantom and for one or more image processing configurations using the radiomics CT phantom. Reviewers may demand that you provide the IBSI compliance spreadsheet for your software.

		matrices were computed.	
CM distance	65	Define the (Chebyshev) distance at which co-occurrence of intensities is determined, e.g. 1.	
SZM linkage distance	66	Define the distance and distance norm for which voxels with the same intensity are considered to belong to the same zone for the purpose of constructing an SZM, e.g. Chebyshev distance of 1.	
DZM linkage distance	67	Define the distance and distance norm for which voxels with the same intensity are considered to belong to the same zone for the purpose of constructing a DZM, e.g. Chebyshev distance of 1.	
DZM zone distance norm	68	Define the distance norm for determining the distance of zones to the border of the ROI, e.g. Manhattan distance.	
NGTDM distance	69	Define the neighbourhood distance and distance norm for the NGTDM, e.g. Chebyshev distance of 1.	
NGLDM distance	70	Define the neighbourhood distance and distance norm for the NGLDM, e.g. Chebyshev distance of 1.	
NGLDM coarseness	71	Define the coarseness parameter for the NGLDM, e.g. 0.	
**Machine learning and radiomics analysis**			
Diagnostic and prognostic modelling	72	See the TRIPOD guidelines for reporting on diagnostic and prognostic modelling.	72 to 75: not applicable
Comparison with known factors	73	Describe where performance of radiomics models is compared with known (clinical) factors.	
Multicollinearity	74	Describe where the multicollinearity between radiomics features in the signature is assessed.	
Model availability	75	Describe where radiomics models with the necessary pre-processing information may be found.	
Data availability	76	Describe where imaging data and relevant meta-data used in the study may be found.	Unfortunately we cannot make image data available for public use.

The reporting guidelines presented above are a copy of the guidelines found in section 4.1 of the IBSI reference manual (see online supplemental materials).
