# Supplementary figures and images for: The role of parametric feature maps to correct different volume of interest sizes: an in vivo liver MRI study
Source: Eur Radiol Exp. 2023 Sep 6;7:48. doi: 10.1186/s41747-023-00362-9 (PMC10480134; doi:10.1186/s41747-023-00362-9)

## Scanner 3 [1.5T] T1w GRE

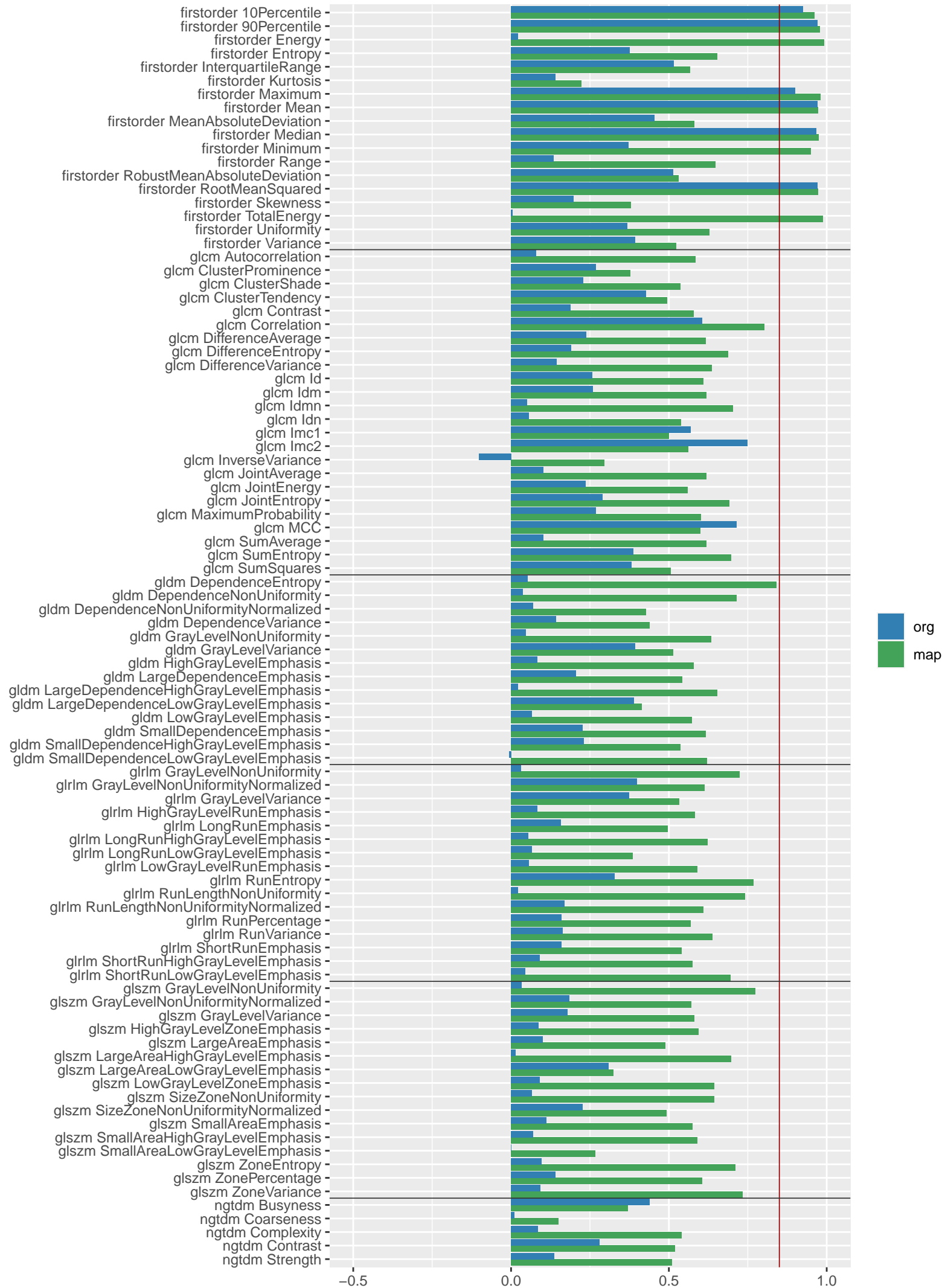

## Scanner 3 [1.5T] T2w TSE

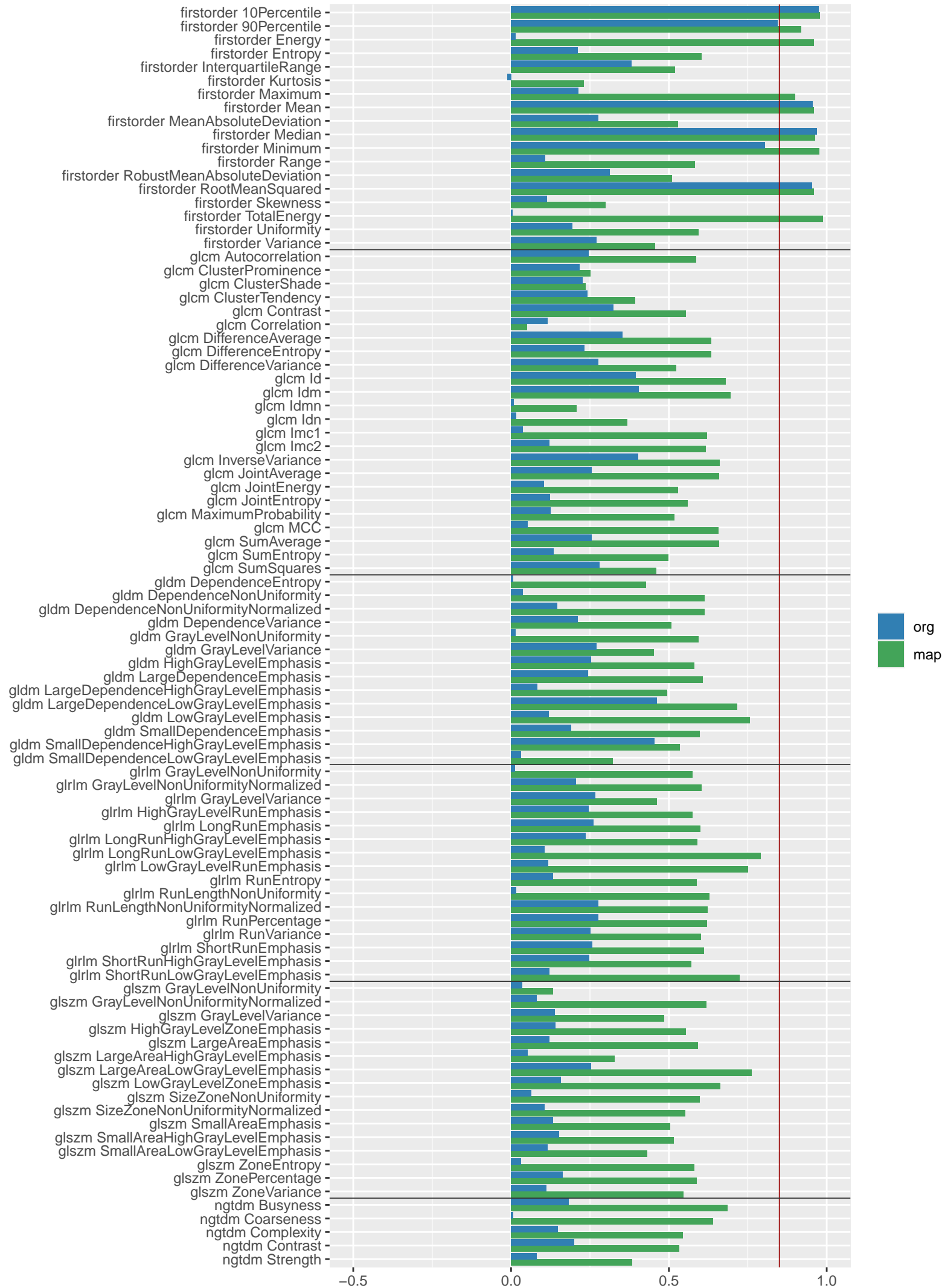

## Scanner 2 [3T] T1w GRE

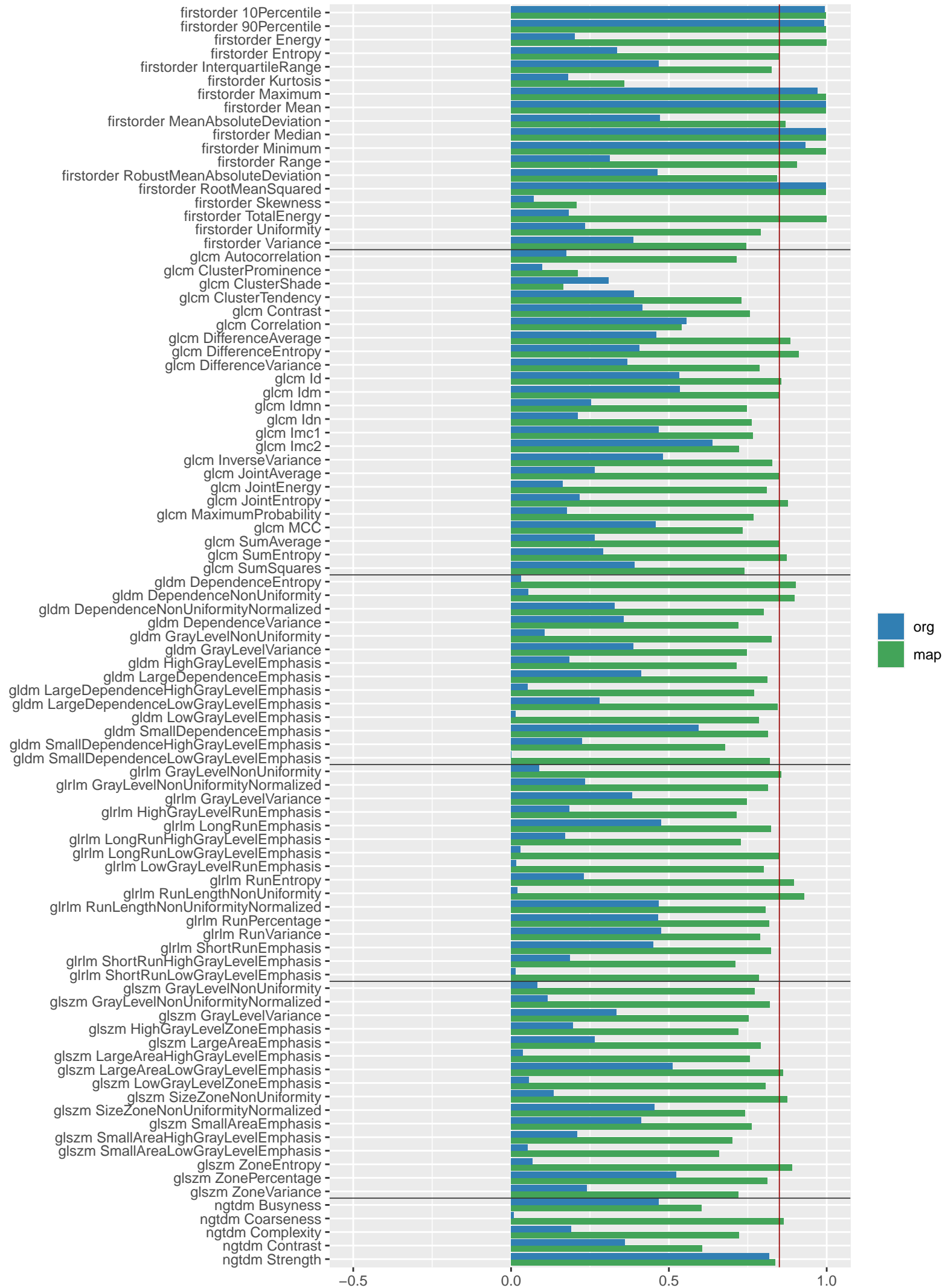

## Scanner 2 [3T] T2w TSE

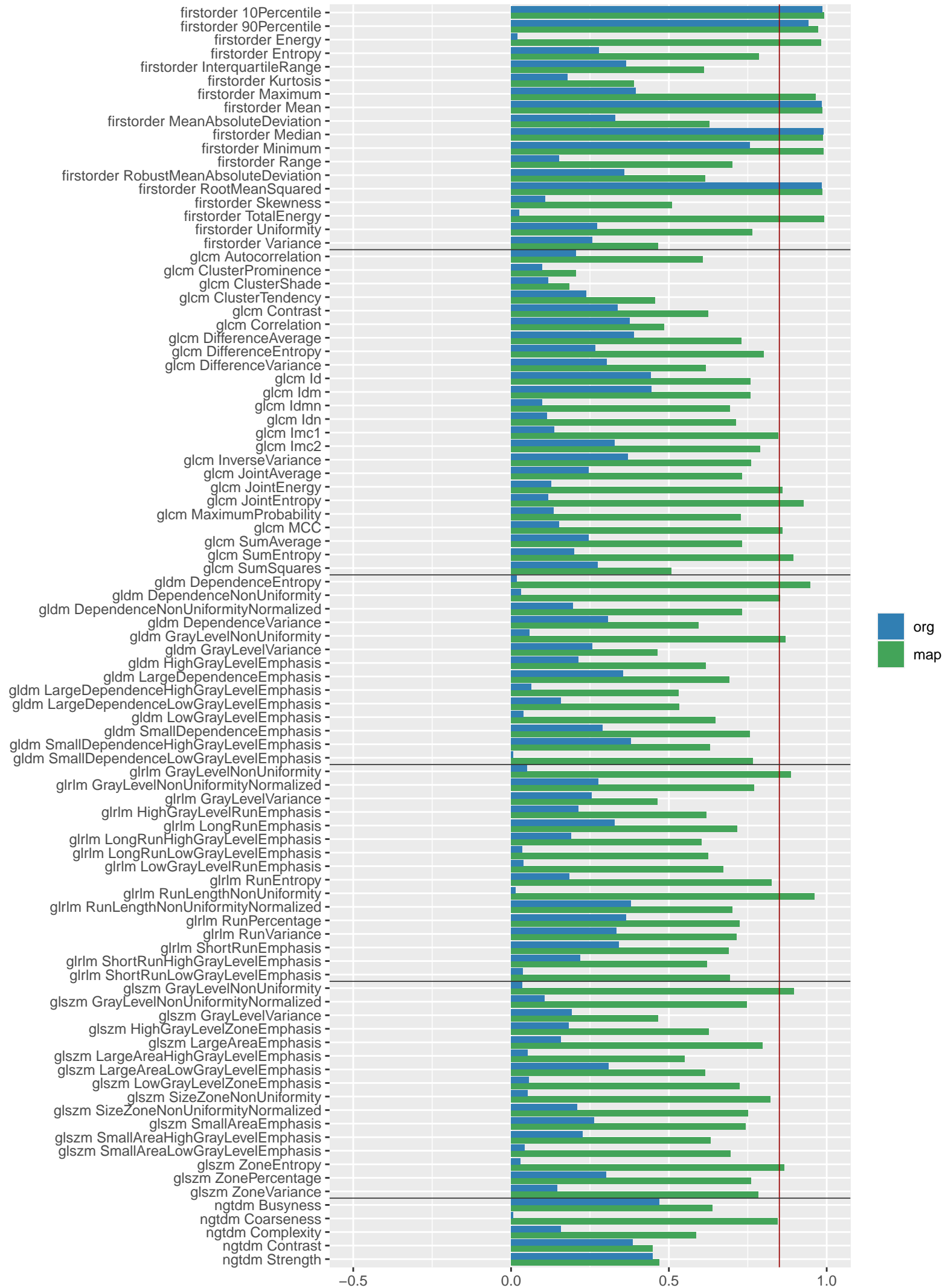

## Scanner 1 [3T] T1w GRE

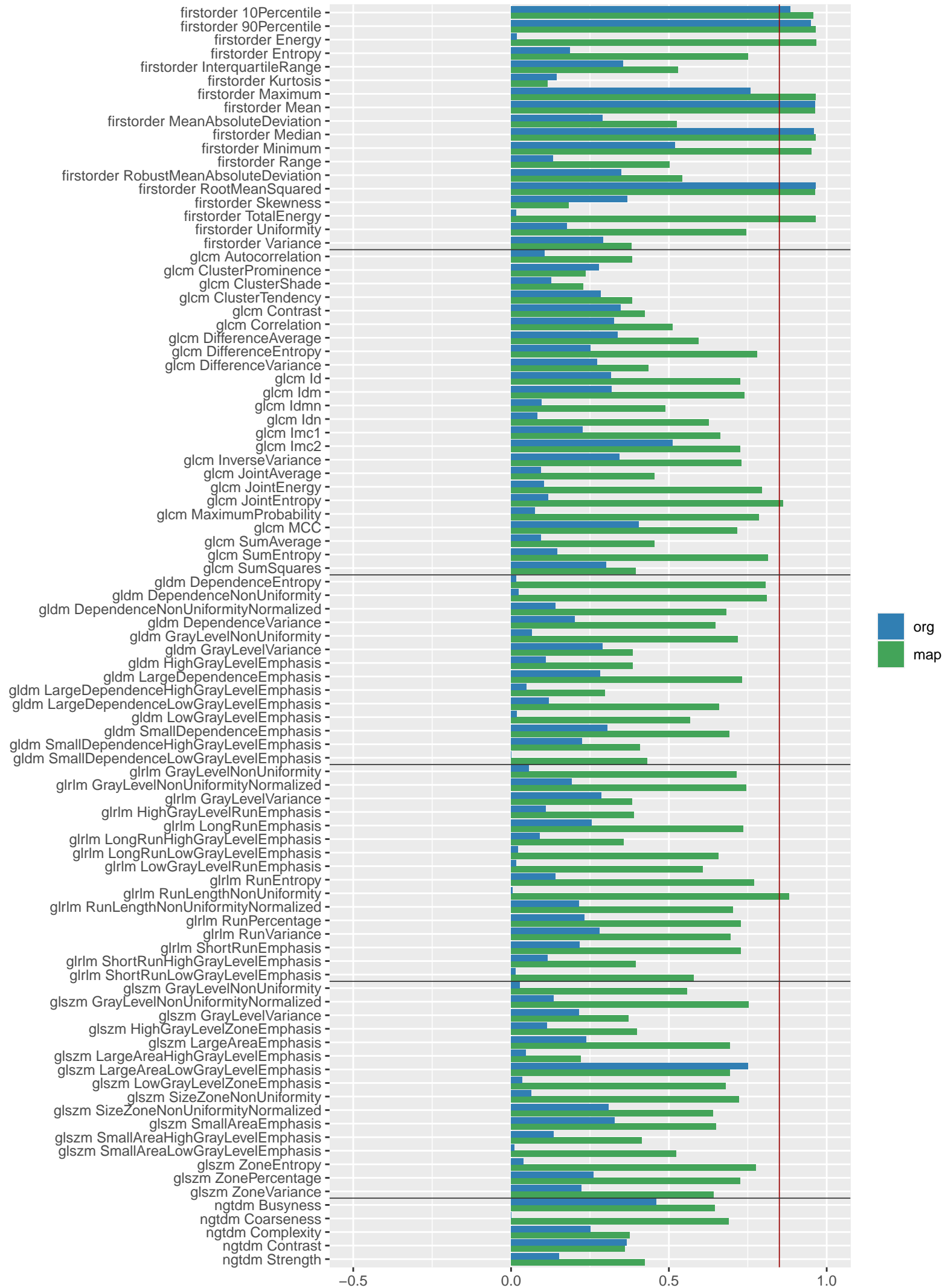

## Scanner 1 [3T] T2w TSE

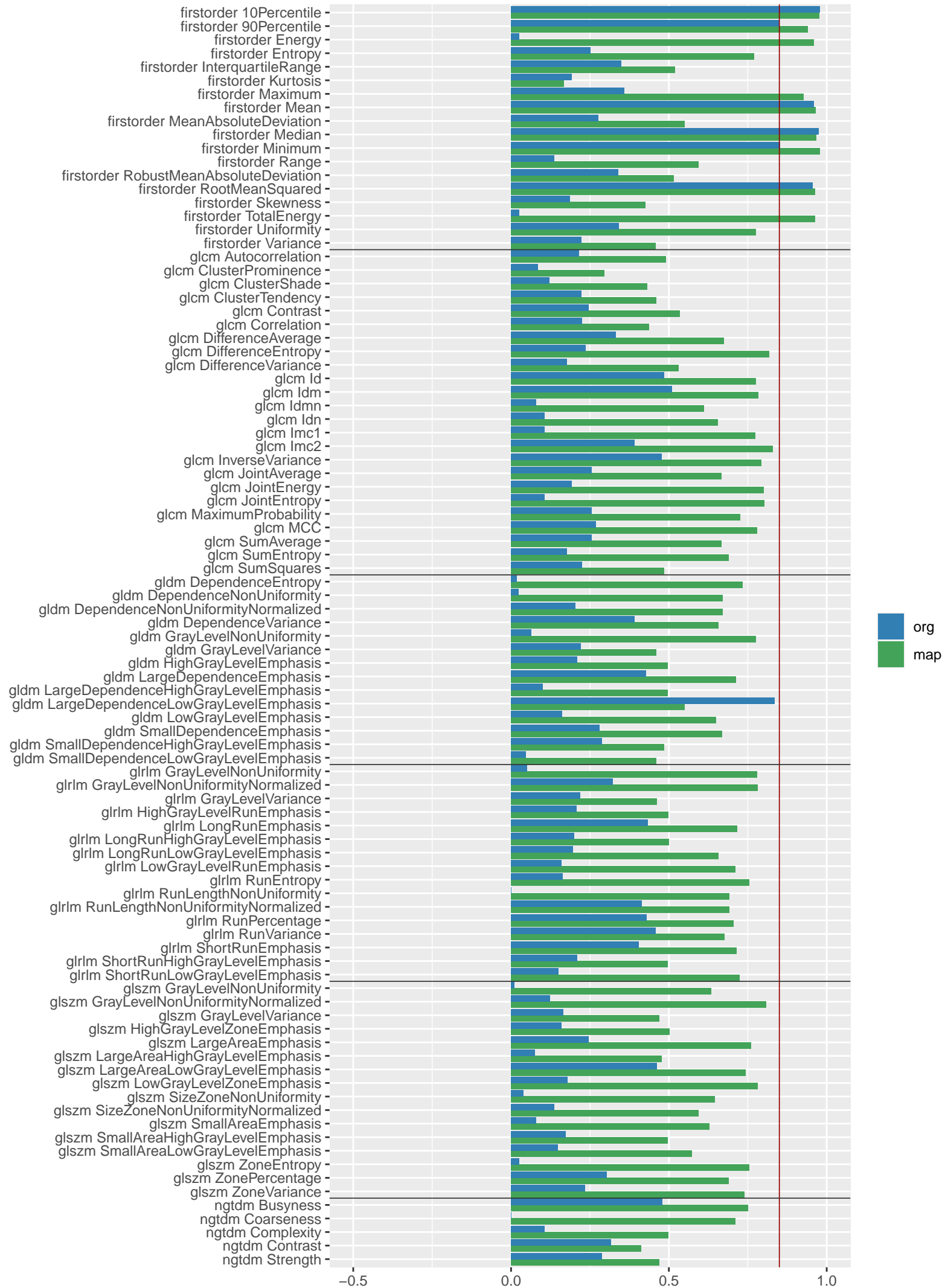

Supplement: Supplementary file 7 — Additional file 7. Barplots of the OCCCs per scanner (VOI sizes of 10, 20, and 30 mm). [file 41747_2023_362_MOESM7_ESM.pdf]

## Scanner 3 [1.5T] T1w GRE

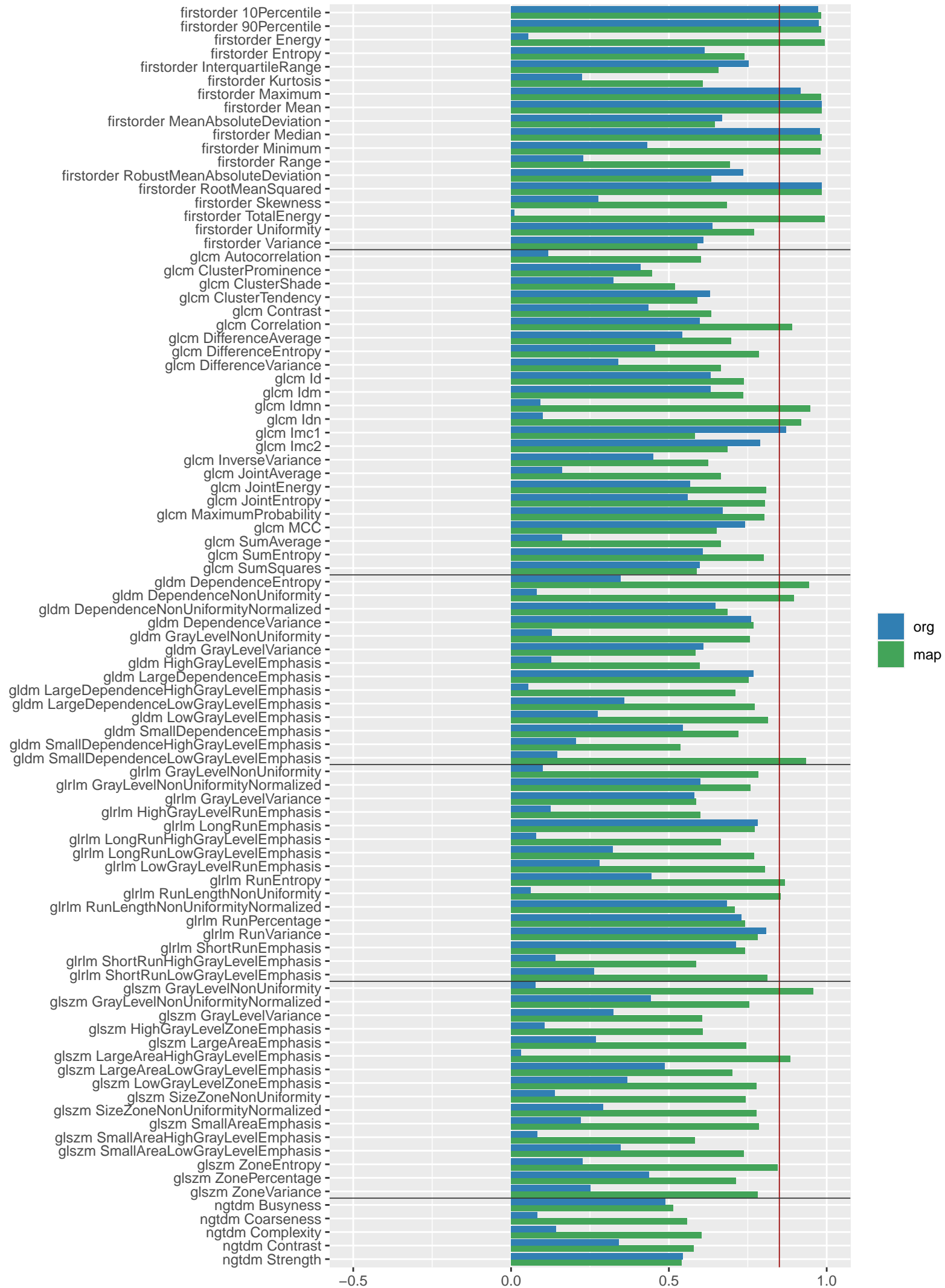

## Scanner 3 [1.5T] T2w TSE

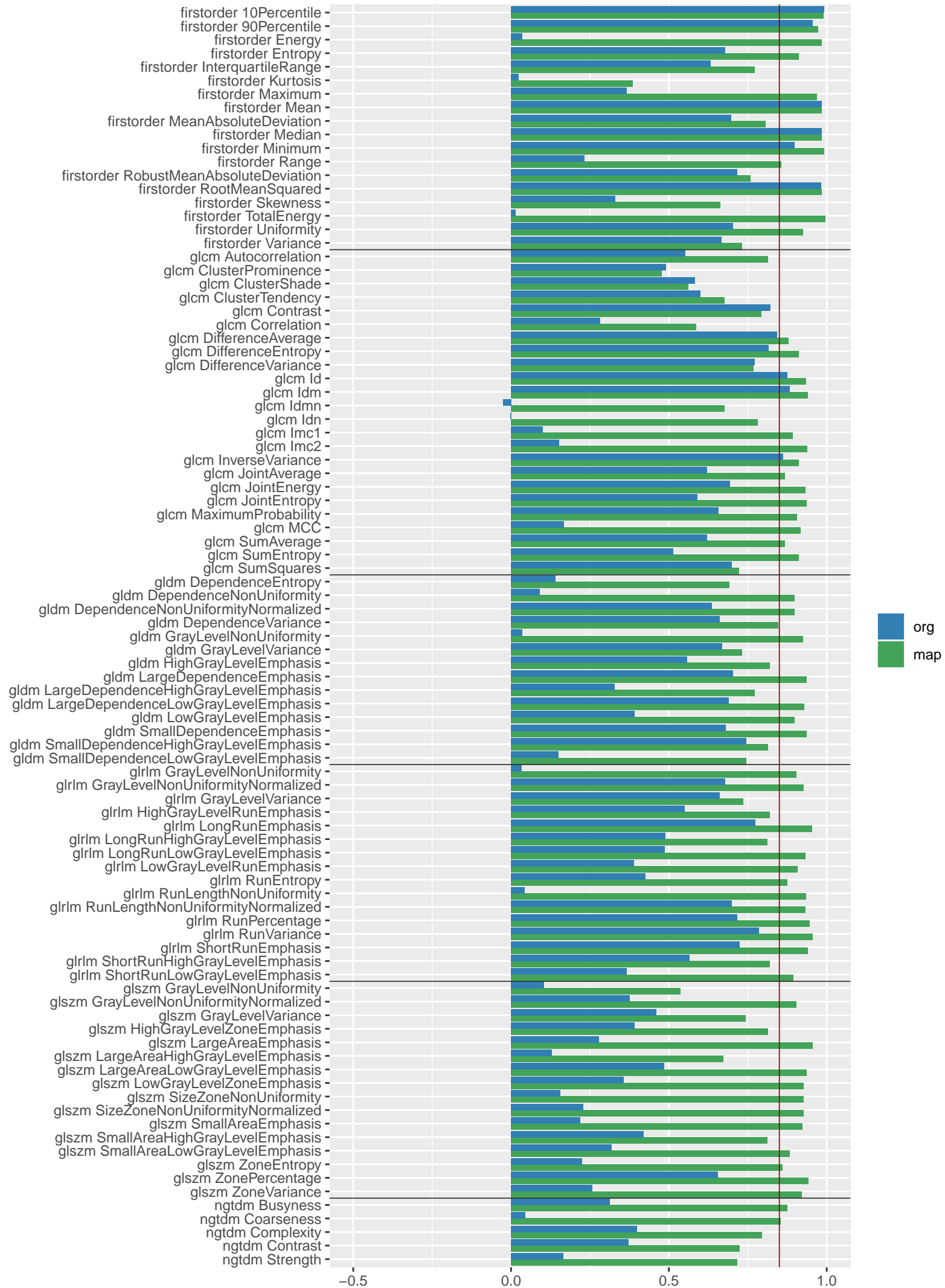

## Scanner 2 [3T] T1w GRE

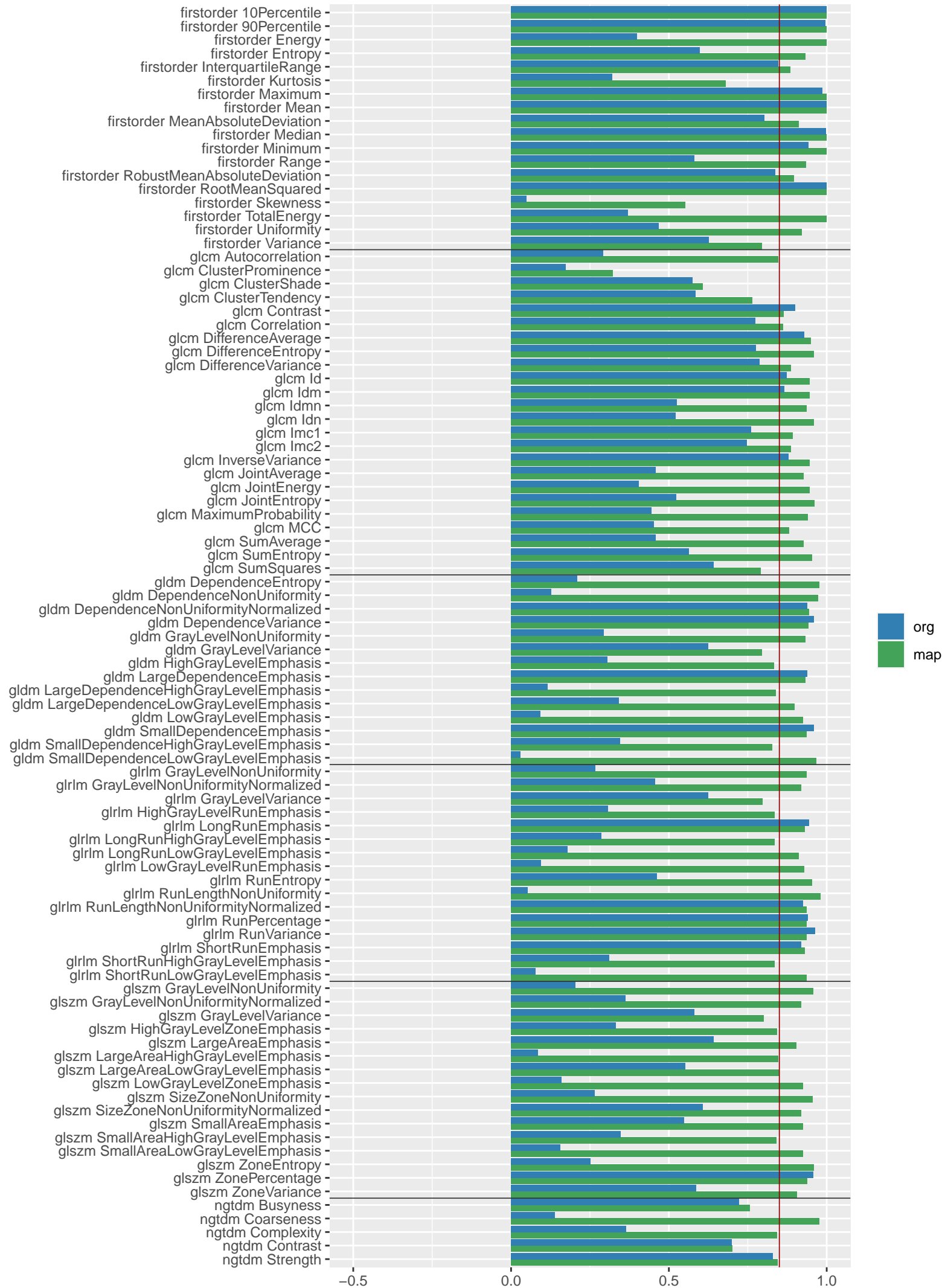

## Scanner 2 [3T] T2w TSE

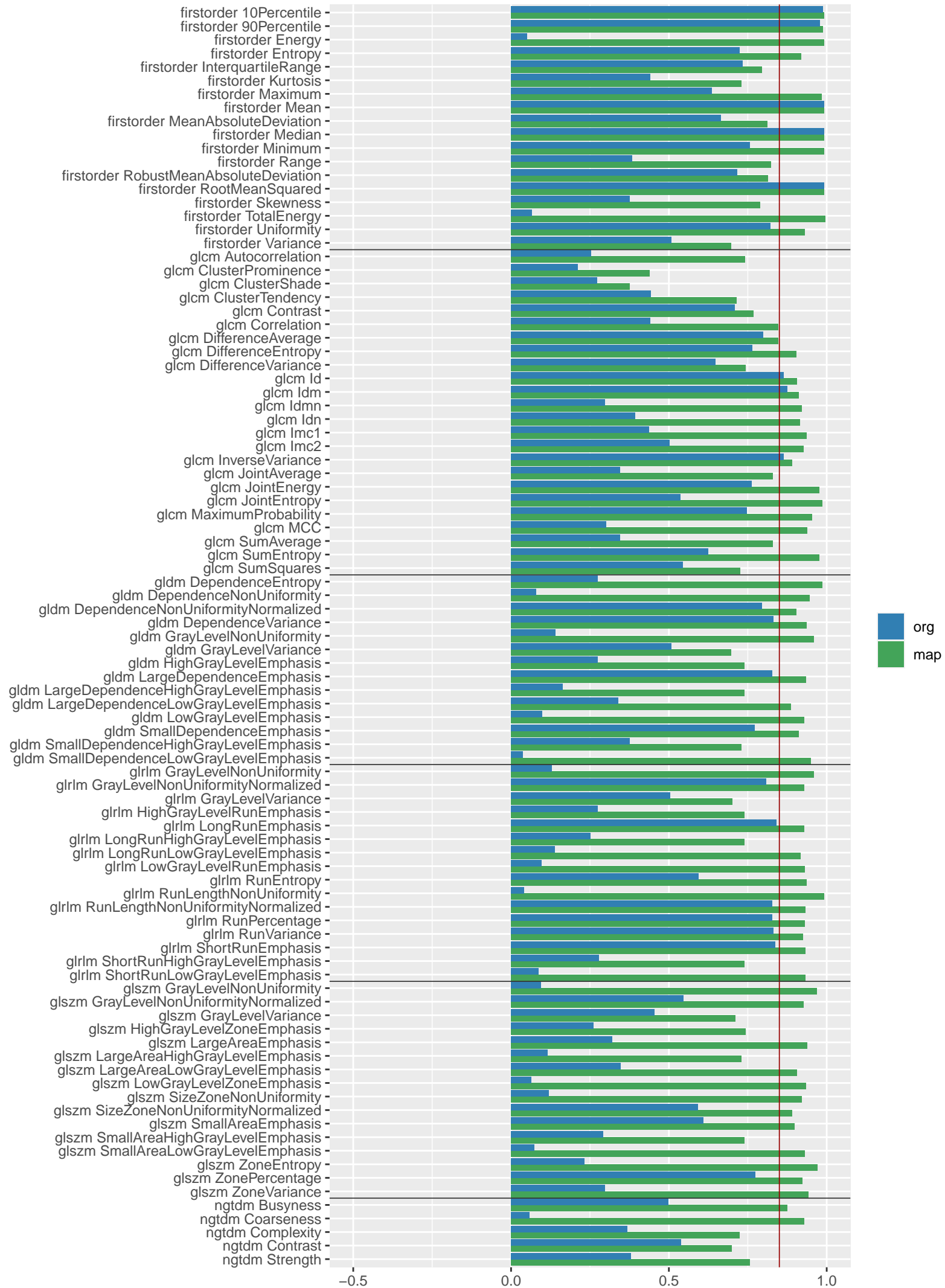

## Scanner 1 [3T] T1w GRE

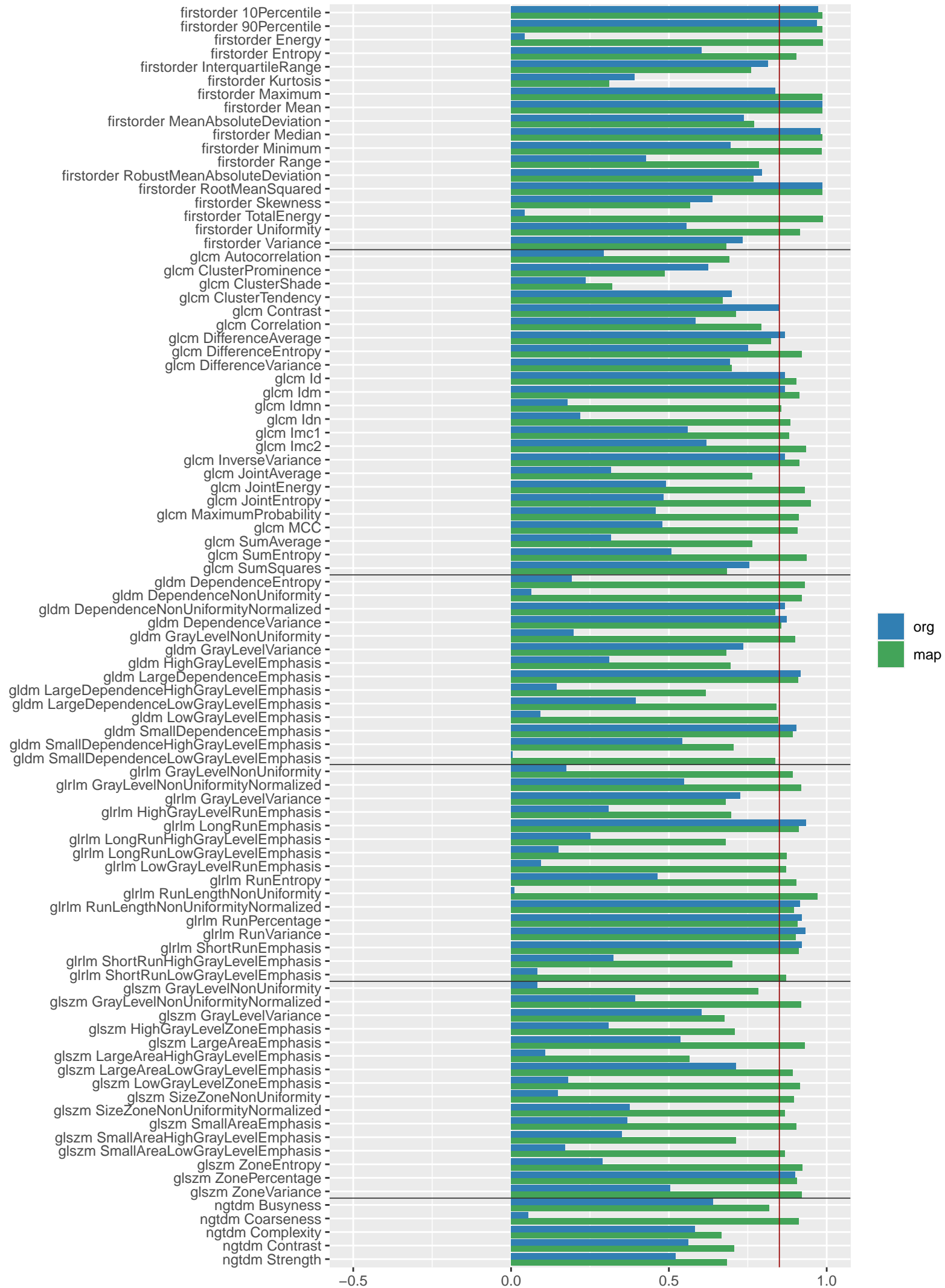

## Scanner 1 [3T] T2w TSE

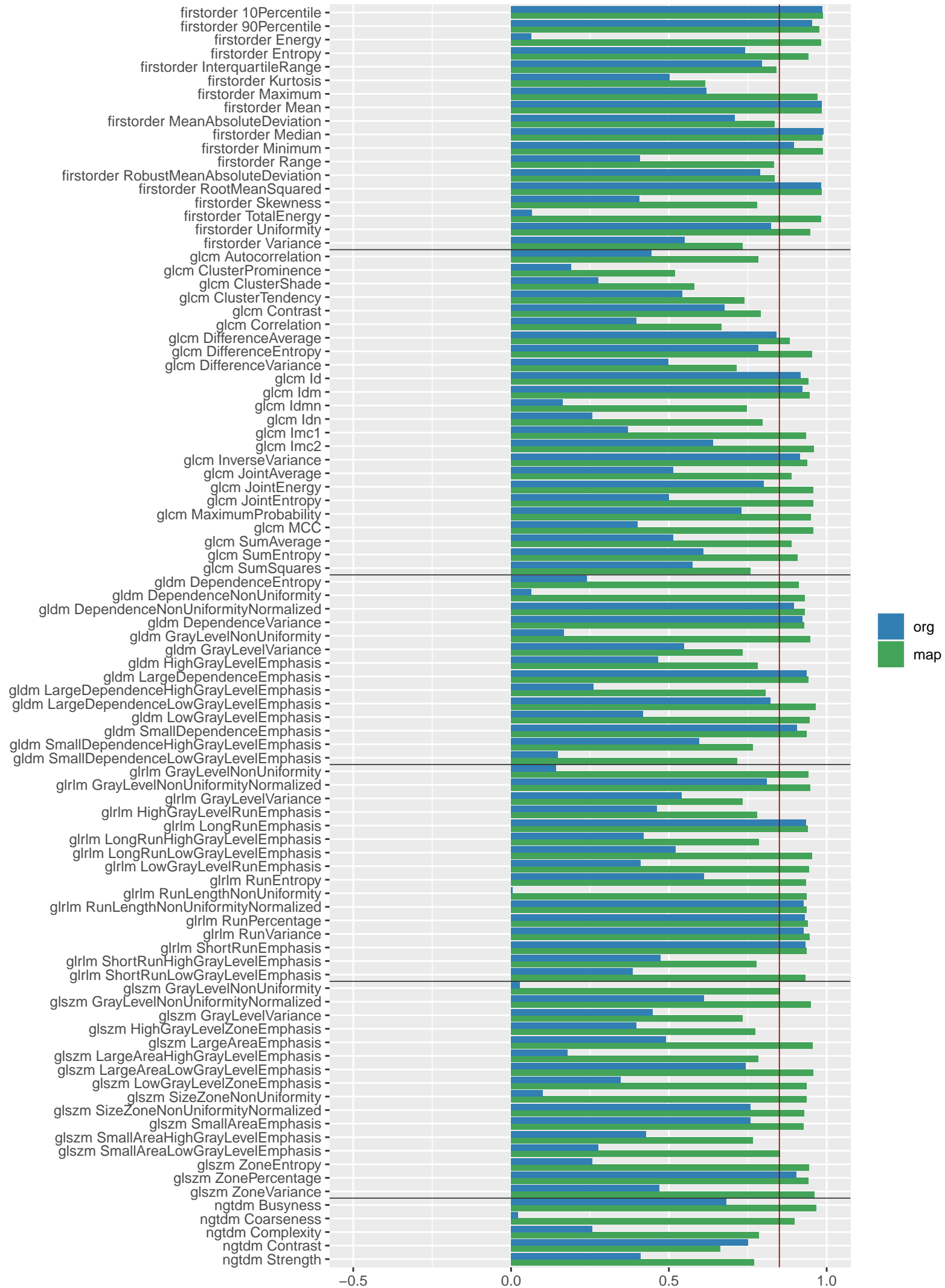

Supplement: Supplementary file 11 — Additional file 11. Barplots of the OCCCs per scanner (VOI sizes of 20 and 30 mm). [file 41747_2023_362_MOESM11_ESM.pdf]
