# Supplementary material for: The role of parametric feature maps to correct different volume of interest sizes: an in vivo liver MRI study
Source: Eur Radiol Exp. 2023 Sep 6;7:48. doi: 10.1186/s41747-023-00362-9 (PMC10480134; doi:10.1186/s41747-023-00362-9)
Supplement: Supplementary file 12 — Additional file 12. Overview of the reproducible features. [file 41747_2023_362_MOESM12_ESM.pdf]

Conventional feature extraction from the original images

1. Across VOI sizes 10, 20, and 30 mm diameter

<table>
<tr><td colspan="3">T1w GRE across VOI sizes 10, 20 mm, and 30 mm diameter – OCCCs ≥ 0.85</td></tr>
<tr><td>Scanner 1</td><td>Scanner 2</td><td>Scanner 3</td></tr>
<tr><td colspan="3">firstorder_RootMeanSquared</td></tr>
<tr><td colspan="3">firstorder_Mean</td></tr>
<tr><td colspan="3">firstorder_Median</td></tr>
<tr><td colspan="3">firstorder_90Percentile</td></tr>
<tr><td colspan="3">firstorder_10Percentile</td></tr>
<tr><td></td><td colspan="2">firstorder_Maximum</td></tr>
<tr><td></td><td>firstorder_Minimum</td><td></td></tr>
</table>

<table>
<tr><td colspan="3">T2w TSE across VOI sizes 10, 20 mm, and 30 mm diameter – OCCCs ≥ 0.85</td></tr>
<tr><td>Scanner 1</td><td>Scanner 2</td><td>Scanner 3</td></tr>
<tr><td colspan="3">firstorder_RootMeanSquared</td></tr>
<tr><td colspan="3">firstorder_Mean</td></tr>
<tr><td colspan="3">firstorder_Median</td></tr>
<tr><td colspan="2">firstorder_90Percentile</td><td></td></tr>
<tr><td colspan="3">10th percentile</td></tr>
<tr><td>firstorder_Minimum</td><td></td><td></td></tr>
</table>

2. Across VOI sizes 20 and 30 mm diameter

T1w GRE across VOI sizes 20 mm and 30 mm diameter – OCCCs ≥ 0.85		
Scanner 1	Scanner 2	Scanner 3
firstorder_RootMeanSquared		
firstorder_Median		
firstorder_Mean		
firstorder_90Percentile		
firstorder_10Percentile		
	firstorder_Maximum	
glcm_Id		
glcm_InverseVariance		
glcm_Contrast		
gldm_DependenceVariance		
gldm_LargeDependenceEmphasis		
gldm_SmallDependenceEmphasis		
gldm_DependenceNonUniformityNormalized		
glrlm_RunVariance		
glrlm_RunPercentage		
glrlm_ShortRunEmphasis		
glrlm_LongRunEmphasis		
glrlm_RunLengthNonUniformityNormalized		
glszm_ZonePercentage		
glcm_DifferenceAverage<br><br>glcm_Idm	firstorder_InterquartileRange<br><br>firstorder_Minimum	glcm_Imc1

T2w TSE across VOI sizes 20 mm and 30 mm diameter – OCCCs ≥ 0.85		
Scanner 1	Scanner 2	Scanner 3
firstorder_RootMeanSquared		
firstorder_Median		
firstorder_Mean		
firstorder_90Percentile		
firstorder_10Percentile		
glcm_Id		
glcm_InverseVariance		
glcm_Idm		
firstorder_Minimum		firstorder_Minimum
glcm_InverseVariance<br>gldm_LargeDependenceEmphasis<br>gldm_DependenceVariance<br>gldm_SmallDependenceEmphasis<br>gldm_DependenceNonUniformityNormalized<br>glrlm_LongRunEmphasis<br>glrlm_ShortRunEmphasis<br>glrlm_RunPercentage<br>glrlm_RunLengthNonUniformityNormalized<br>glrlm_RunVariance<br>glszm_ZonePercentage		

Direct feature extraction from the parametric maps

1. Across VOI sizes 10, 20, and 30 mm diameter

<table>
<tr><td colspan="3">T1w GRE across VOI sizes 10, 20 mm, and 30 mm diameter – OCCCs ≥ 0.85</td></tr>
<tr><td>Scanner 1</td><td>Scanner 2</td><td>Scanner 3</td></tr>
<tr><td colspan="3">firstorder_RootMeanSquared</td></tr>
<tr><td colspan="3">firstorder_Mean</td></tr>
<tr><td colspan="3">firstorder_Median</td></tr>
<tr><td colspan="3">firstorder_90Percentile</td></tr>
<tr><td colspan="3">firstorder_10Percentile</td></tr>
<tr><td colspan="3">firstorder_Maximum</td></tr>
<tr><td colspan="3">firstorder_Minimum</td></tr>
<tr><td colspan="3">firstorder_Energy</td></tr>
<tr><td colspan="3">firstorder_TotalEnergy</td></tr>
<tr><td colspan="2">glrlm_RunLengthNonUniformity</td><td></td></tr>
<tr><td colspan="2">glcm_JointEntropy</td><td></td></tr>
<tr><td></td><td>firstorder_Range
firstorder_MeanAbsoluteDeviation
firstorder_Entropy
glcm_DifferenceEntropy
glcm_DifferenceAverage
glcm_SumEntropy
glcm_Id
glcm_Idm
glcm_SumAverage
glcm_JointAverage
gldm_LargeDependenceLowGrayLevelEmphasis
gldm_DependenceEntropy
gldm_DependenceNonUniformity
glrlm_RunEntropy
glrlm_GrayLevelNonUniformity
glrlm_LongRunLowGrayLevelEmphasis</td><td></td></tr>
</table>

	glszm_LargeAreaLowGrayLevelEmphasis<br><br>glszm_ZoneEntropy<br><br>glszm_SizeZoneNonUniformity<br><br>ngtdm_Coarseness	

T2w TSE across VOI sizes 10, 20 mm, and 30 mm diameter – OCCCs ≥ 0.85		
Scanner 1	Scanner 2	Scanner 3
firstorder_RootMeanSquared		
firstorder_Mean		
firstorder_Median		
firstorder_90Percentile		
firstorder_10Percentile		
firstorder_Maximum		
firstorder_Minimum		
firstorder_Energy		
firstorder_TotalEnergy		
	glcm_Imc1<br><br>glcm_JointEntropy<br><br>glcm_SumEntropy<br><br>glcm_MCC<br><br>glcm_JointEnergy<br><br>gldm_GrayLevelNonUniformity<br><br>gldm_DependenceEntropy<br><br>gldm_DependenceNonUniformity<br><br>glrlm_RunLengthNonUniformity<br><br>glrlm_GrayLevelNonUniformity<br><br>glszm_GrayLevelNonUniformity<br><br>glszm_ZoneEntropy<br><br>ngtdm_Coarseness	

2. Across VOI sizes 20 and 30 mm diameter

<table>
<tr><td colspan="3">T1w GRE across VOI sizes 20 mm and 30 mm diameter – OCCCs ≥ 0.85</td></tr>
<tr><td>Scanner 1</td><td>Scanner 2</td><td>Scanner 3</td></tr>
<tr><td colspan="3">firstorder_RootMeanSquared</td></tr>
<tr><td colspan="3">firstorder_Mean</td></tr>
<tr><td colspan="3">firstorder_Median</td></tr>
<tr><td colspan="3">firstorder_90Percentile</td></tr>
<tr><td colspan="3">firstorder_10Percentile</td></tr>
<tr><td colspan="3">firstorder_Maximum</td></tr>
<tr><td colspan="3">firstorder_Minimum</td></tr>
<tr><td colspan="3">firstorder_Energy</td></tr>
<tr><td colspan="3">firstorder_TotalEnergy</td></tr>
<tr><td colspan="3">glcm_Idm</td></tr>
<tr><td colspan="3">glcm_Idn</td></tr>
<tr><td colspan="3">gldm_DependenceEntropy</td></tr>
<tr><td colspan="3">gldm_DependenceNonUniformity</td></tr>
<tr><td colspan="3">glrlm_RunEntropy</td></tr>
<tr><td colspan="3">glrlm_RunLengthNonUniformity</td></tr>
<tr><td colspan="3">glszm_ZoneEntropy</td></tr>
<tr><td></td><td colspan="2">glcm_Correlation</td></tr>
<tr><td></td><td colspan="2">gldm_SmallDependenceLowGrayLevelEmphasis</td></tr>
<tr><td></td><td colspan="2">glszm_GrayLevelNonUniformity</td></tr>
<tr><td></td><td colspan="2">glszm_LargeAreaHighGrayLevelEmphasis</td></tr>
<tr><td colspan="2">firstorder_Uniformity</td><td></td></tr>
<tr><td colspan="2">firstorder_Entropy</td><td></td></tr>
<tr><td colspan="2">glcm_JointEntropy</td><td></td></tr>
<tr><td colspan="2">glcm_Id</td><td></td></tr>
<tr><td colspan="2">glcm_SumEntropy</td><td></td></tr>
<tr><td colspan="2">glcm_Imc2</td><td></td></tr>
<tr><td colspan="2">glcm_JointEnergy</td><td></td></tr>
<tr><td colspan="2">glcm_DifferenceEntropy</td><td></td></tr>
</table>

glcm_InverseVariance	
glcm_MaximumProbability	
glcm_MCC	
glcm_Imc1	
glcm_Idmn	
gldm_LargeDependenceEmphasis	
gldm_GrayLevelNonUniformity	
gldm_SmallDependenceEmphasis	
gldm_DependenceVariance	
gldm_LowGrayLevelEmphasis	
glrlm_GrayLevelNonUniformityNormalized	
glrlm_LongRunEmphasis	
glrlm_ShortRunEmphasis	
glrlm_RunPercentage	
glrlm_RunVariance	
glrlm_RunLengthNonUniformityNormalized	
glrlm_GrayLevelNonUniformity	
glrlm_LongRunLowGrayLevelEmphasis	
glrlm_ShortRunLowGrayLevelEmphasis	
glrlm_LowGrayLevelRunEmphasis	
glszm_SmallAreaLowGrayLevelEmphasis	
glszm_LargeAreaEmphasis	
glszm_ZoneVariance	
glszm_GrayLevelNonUniformityNormalized	
glszm_LowGrayLevelZoneEmphasis	
glszm_ZonePercentage	
glszm_SmallAreaEmphasis	
glszm_SizeZoneNonUniformity	
glszm_LargeAreaLowGrayLevelEmphasis	
glszm_SizeZoneNonUniformityNormalized	
ngtdm_Coarseness	

	firstorder_MeanAbsoluteDeviation<br>firstorder_RobustMeanAbsoluteDeviation<br>firstorder_InterquartileRange<br>firstorder_Range<br>glcm_JointAverage<br>glcm_SumAverage<br>glcm_DifferenceAverage<br>glcm_DifferenceVariance<br>glcm_Contrast<br>glcm_Autocorrelation<br>gldm_DependenceNonUniformityNormalized<br>gldm_LargeDependenceLowGrayLevelEmphasis<br>ngtdm_Strength	

T2w TSE across VOI sizes 20 mm and 30 mm diameter – OCCCs ≥ 0.85		
Scanner 1	Scanner 2	Scanner 3
firstorder_RootMeanSquared		
firstorder_Mean		
firstorder_Median		
firstorder_10Percentile		
firstorder_Maximum		
firstorder_Minimum		
firstorder_Energy		
firstorder_TotalEnergy		
firstorder_Uniformity		
firstorder_Entropy		
glcm_Imc2		
glcm_JointEnergy		
glcm_JointEntropy		
glcm_MCC		
glcm_DifferenceEntropy		
glcm_MaximumProbability		
glcm_Idm		
glcm_Id		
glcm_InverseVariance		
glcm_Imc1		
glcm_SumEntropy		
glcm_DifferenceAverage		
gldm_LargeDependenceLowGrayLevelEmphasis		
gldm_GrayLevelNonUniformity		
gldm_LowGrayLevelEmphasis		
gldm_LargeDependenceEmphasis		
gldm_SmallDependenceEmphasis		
gldm_DependenceNonUniformity		
gldm_DependenceNonUniformityNormalized		

gldm_DependenceVariance		
glrlm_LongRunLowGrayLevelEmphasis		
glrlm_RunVariance		
glrlm_LowGrayLevelRunEmphasis		
glrlm_GrayLevelNonUniformity		
glrlm_GrayLevelNonUniformityNormalized		
glrlm_LongRunEmphasis		
glrlm_RunPercentage		
glrlm_RunLengthNonUniformity		
glrlm_RunLengthNonUniformityNormalized		
glrlm_ShortRunEmphasis		
glrlm_RunEntropy		
glrlm_ShortRunLowGrayLevelEmphasis		
glszm_ZoneVariance		
glszm_LargeAreaLowGrayLevelEmphasis		
glszm_LargeAreaEmphasis		
glszm_GrayLevelNonUniformityNormalized		
glszm_ZoneEntropy		
glszm_ZonePercentage		
glszm_SizeZoneNonUniformity		
glszm_LowGrayLevelZoneEmphasis		
glszm_SizeZoneNonUniformityNormalized		
glszm_SmallAreaEmphasis		
glszm_SmallAreaLowGrayLevelEmphasis		
ngtdm_Busyness		
ngtdm_Coarseness		
	glcm_Idn	
glszm_GrayLevelNonUniformity		
gldm_DependenceEntropy		
firstorder_90Percentile<br>glcm_JointAverage		firstorder_90Percentile<br>glcm_JointAverage

glcm_SumAverage		glcm_SumAverage
	gldm_SmallDependenceLowGrayLevelEmphasis<br><br>glcm_Idmn<br><br>glcm_Correlation	firstorder_Range
